# The Dissection of Nitrogen Response Traits Using Drone Phenotyping and Dynamic Phenotypic Analysis to Explore N Responsiveness and Associated Genetic Loci in Wheat

**DOI:** 10.34133/plantphenomics.0128

**Published:** 2023-12-22

**Authors:** Guohui Ding, Liyan Shen, Jie Dai, Robert Jackson, Shuchen Liu, Mujahid Ali, Li Sun, Mingxing Wen, Jin Xiao, Greg Deakin, Dong Jiang, Xiu-e Wang, Ji Zhou

**Affiliations:** ^1^ College of Agriculture, Plant Phenomics Research Centre, Academy for Advanced Interdisciplinary Studies, Nanjing Agricultural University, Nanjing 210095, China.; ^2^ Cambridge Crop Research, National Institute of Agricultural Botany (NIAB), Cambridge CB3 0LE, UK.; ^3^ State Key Laboratory of Crop Genetics and Germplasm Enhancement, Cytogenetics Institute, Nanjing Agricultural University/JCIC-MCP, Nanjing, Jiangsu 210095, China.; ^4^ Zhenjiang Institute of Agricultural Science, Jurong, Jiangsu 212400, China.; ^5^ Regional Technique Innovation Center for Wheat Production, Key Laboratory of Crop Physiology and Ecology in Southern China, Ministry of Agriculture, Nanjing Agricultural University, Nanjing, Jiangsu 210095, China.

## Abstract

Inefficient nitrogen (N) utilization in agricultural production has led to many negative impacts such as excessive use of N fertilizers, redundant plant growth, greenhouse gases, long-lasting toxicity in ecosystem, and even effect on human health, indicating the importance to optimize N applications in cropping systems. Here, we present a multiseasonal study that focused on measuring phenotypic changes in wheat plants when they were responding to different N treatments under field conditions. Powered by drone-based aerial phenotyping and the AirMeasurer platform, we first quantified 6 N response-related traits as targets using plot-based morphological, spectral, and textural signals collected from 54 winter wheat varieties. Then, we developed dynamic phenotypic analysis using curve fitting to establish profile curves of the traits during the season, which enabled us to compute static phenotypes at key growth stages and dynamic phenotypes (i.e., phenotypic changes) during N response. After that, we combine 12 yield production and N-utilization indices manually measured to produce N efficiency comprehensive scores (NECS), based on which we classified the varieties into 4 N responsiveness (i.e., N-dependent yield increase) groups. The NECS ranking facilitated us to establish a tailored machine learning model for N responsiveness-related varietal classification just using N-response phenotypes with high accuracies. Finally, we employed the Wheat55K SNP Array to map single-nucleotide polymorphisms using N response-related static and dynamic phenotypes, helping us explore genetic components underlying N responsiveness in wheat. In summary, we believe that our work demonstrates valuable advances in N response-related plant research, which could have major implications for improving N sustainability in wheat breeding and production.

## Infographic

**Figure:**
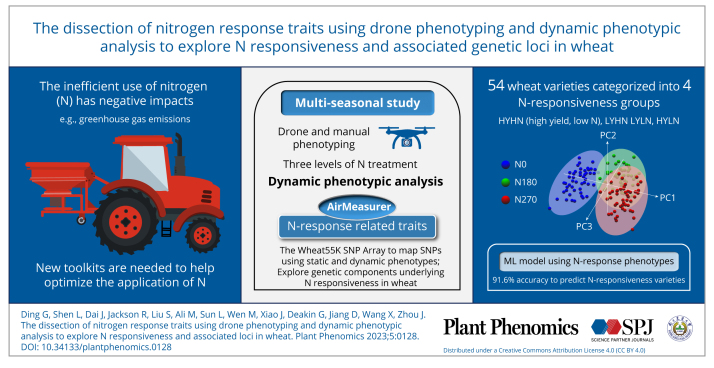

## Introduction

The imminent challenges of climate change, growing food demand, and fertilizer shortage have brought enormous threats to global food security [1]. As one of the most consumed grains in the world, wheat (*Triticum aestivum* L.) and its sustainable production are paramount to ensure food supply globally [2]. Modern wheat production heavily relies on synthetic nitrogen (N) fertilizers to reach yield potential, underpinning grain production and quality [3,4]. Nevertheless, inefficient N utilization in agricultural production could lead to many negative impacts, including the excessive use of N fertilizers, redundant crop growth (e.g., making plants more vulnerable to insects and diseases), greenhouse gases emission, long-lasting toxicity in ecosystem, and even effect on human health such as thyroid conditions and diabetes [5–8]. Hence, it is imperative to seek ways to optimize N applications, which include: (a) enhanced N management, involving N testing in the soil, N applications in terms of rates and timing, and crop rotation [9]; (b) precision agricultural activities, identifying temporal and spatial variations in N content across the field to promote effective use of N [10]; (c) variety selection, seeking crop varieties with better N use efficiency (NUE) performance [11,12].

Recent advances in molecular breeding and genetic engineering demonstrate new approaches to improve NUE and N utilization in cropping system, helping breeders and plant researchers assess N responsiveness (i.e., N-dependent yield increase) in wheat and thus apply marker-assisted selection to develop desirable crop varieties [13,14]. Nevertheless, problems associated with these approaches still need to be addressed, including: (a) genetic complexity: it is difficult to dissect the wheat genome into smaller and defined segments, preventing us from fully understanding the genetics of NUE and N responsiveness as they are regulated by the expression of multiple genes and their interactions [15,16]; (b) phenotypic variation: N response-related traits are influenced by environmental factors, soil fertility, and agronomic practices, making it difficult to compare target traits and associated phenotypes under field conditions [17]; (c) experimental variability: inconsistent experimental settings and phenotyping methods across multiple seasons and sites could cause problematic integration and comparison of indices such as N-utilization efficiency, N-uptake efficiency (NupE), and N harvest index (NHI), leading to inconclusive findings [18,19]. Among the problems listed above, the ability to characterize N response-related traits in a consistent manner forms the foundation of reproducibly measuring dynamic phenotypic changes during N response, which is key to the reliable association of underlying genetic components relevant to N responsiveness in wheat.

In order to develop reproducible data collection and trait analysis under varied agronomic inputs, the rapid development of unmanned aerial vehicles (e.g., drones) technologies and in-field aerial phenotyping proved to be valuable [20,21]. Due to decreasing costs of remote sensors such as red-green-blue cameras, multi- and hyperspectral devices, and thermal sensors [22–24], aerial phenotyping has been popularly employed to assess plant’s yield- and performance-related traits in the field. For example, millions of iceberg lettuces were quantified based on high-definition normalized difference vegetation index imagery collected by a light aircraft to evaluate marketable yield [25]; drone-acquired multiple vegetation indices were employed to study crop performance in rice using hyperspectral and red-green-blue image sensors [26]; multimodal data fusion was applied to analyze multispectral imagery for yield prediction in soybean [27]; dynamic features of growth-related traits were quantified from 2-dimensional (2D)/3-dimensional (3D) aerial image series, which also led to reliable genetic loci in paddy rice [28]; aerial thermal images were employed to map crops such as corn and barley so that vegetation phases and soil temperature could be monitored during the season [29]; supervised machine learning (ML) models were proposed to estimate field-level spatial distribution and aboveground biomass in maize based on drone-collected imagery [30].

In wheat, several case studies have been reported in using N response-related patterns to examine N responsiveness and NUE features, including (a) site-specific N management was enabled by aerial phenotyping to assess plant canopy’s N status to estimate available N content in the soil and thus high NUE genotypes [31]; (b) 3D point clouds were used to measure canopy structural differences to classify varietal NUE performance [32]; (c) vegetative indices measured under different N applications were used to determine optimized N requirement [33]; a range of growth traits such as plant height [34], leaf area index [35], leaf greenness [36], phenology [37], and spike number [38] were employed to derive N utilization-related indices such as N-utilization efficiency, NupE, and NHI, facilitating studies on N uptake, N utilization, N responsiveness, and N management. Nevertheless, much research focused on specific traits or proxies measured at limited time points during the season, which overly simplified the dynamic feature of N response and related phenotypic changes [22,39]. In a trial containing diverse genotypes, different lines developed at dissimilar paces, requiring a more systematic and vigorous approach to pinpoint desired developmental patterns [40,41]. Hence, some researchers [16,42,43] indicated that the integration of multiple N-response traits was likely to identify desired NUE and N-responsiveness features, which could help reveal fundamental molecular regulation of N utilization. Still, how to quantify temporal changes of target traits and their relations to yield production requires new toolkits that are capable of integrating N supply, N utilization, N response phenotypes, and yield performance into a reproducible approach to address the above challenges.

Here, we present a study that focuses on establishing dynamic phenotypic analysis to quantify N-response phenotypes based on drone phenotyping. First, we performed aerial phenotyping to acquire plot-level wheat canopy images in a multiseasonal field experiment. Based on morphological, spectral, and textural signals collected from 54 wheat varieties under 3 N treatments (3 replicates, 486 plots per season), we employed the AirMeasurer platform [28] to derive 6 N response-related traits or proxies as targets (hereinafter traits). Then, using static phenotypes measured at 10 time points when drone phenotyping was conducted, we developed dynamic phenotypic analysis with varied curve fitting functions, resulting in profile curves for each trait. These curves enabled us to quantify phenotypic changes (i.e., dynamic phenotypes) during N response. Moreover, we applied principal component analysis (PCA) to examine 12 yield-performance and N-utilization (Y&N) indices, classifying the varieties into 4 N-responsiveness groups. To integrate Y&N indices and N-response phenotypes into a unified approach to assess N responsiveness in wheat, a supervised ML model called random forest-nitrogen response (RF-NRES) was established, which was able to identify wheat varieties’ N responsiveness features just using varietal phenotypic changes. Finally, we explored the use of static and dynamic phenotypes in genetic mapping, showing the value of dynamic phenotyping in associating N-response phenotypes with underlying genetic loci. We believe that our work demonstrates valuable advances in N response-related phenotypic analysis, which could have substantial implications in studying N-responsiveness features, associated genetic components, as well as facilitating the improvement of N sustainability in wheat breeding and production.

## Materials and Methods

### Plant materials and field experiments

We collected 105 representative winter wheat varieties from the middle and lower reaches of the Yangtze River and other main wheat production regions in China. The Zhenjiang Agricultural Science and Technology Innovation Center (31°57’N, 119°18’E; Jiangsu China) was chosen to conduct a 3-year field experiment due to its stable weather condition. The first season (2018 to 2019) was designed to screen the 105 varieties, during which they were studied in 1-m^2^ plots (1 × 1 m), and their NUE scores were calculated using the equation $\frac{\mathrm{GY}}{N_{s}}$, where *GY* is total grain yield and *N_s_* is total N supply [44]. Then, we selected 54 varieties (Table S1) with similar developmental phases and better NUE scores in the following trials in the 2019 to 2021 seasons.

**Fig. 1.**
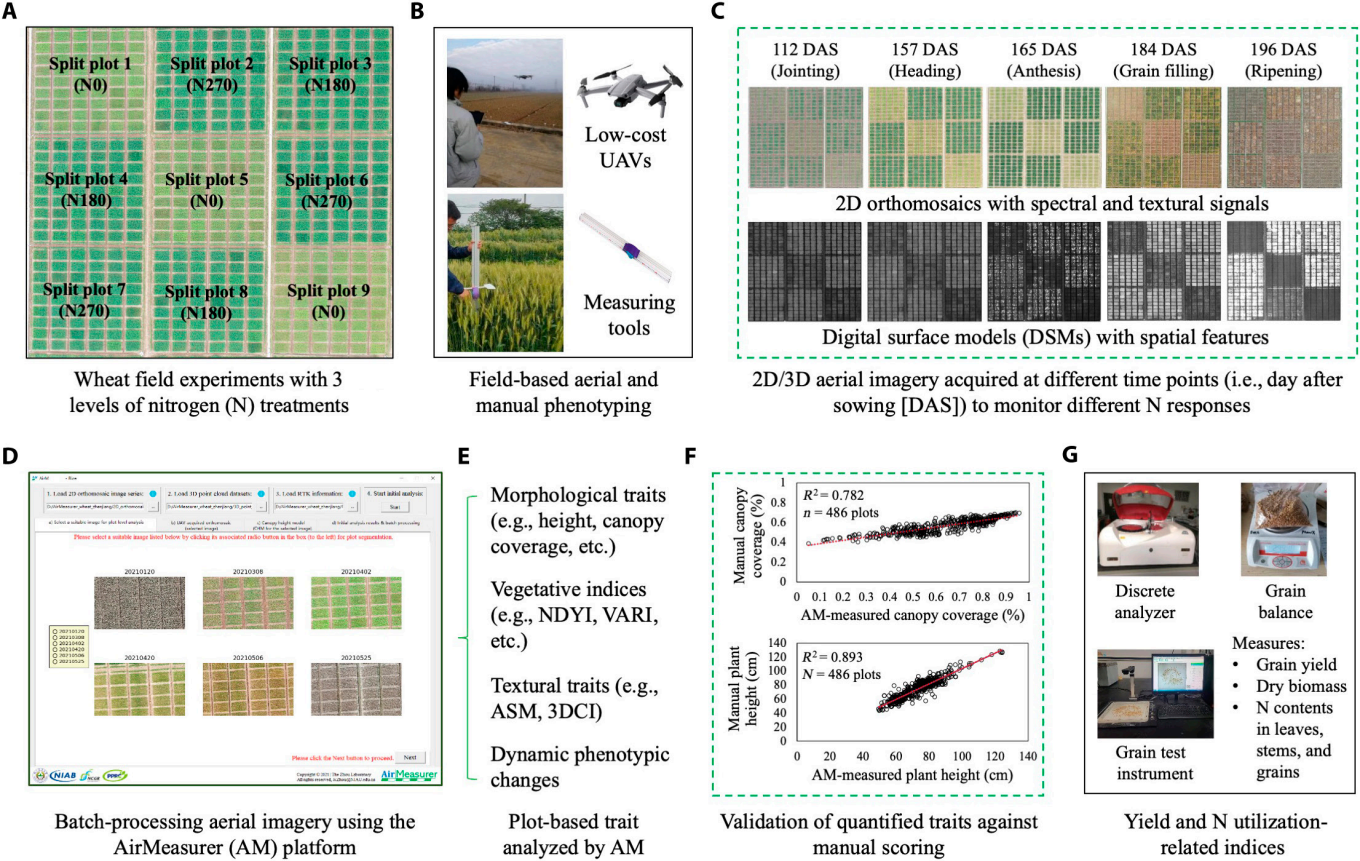
Drone and manual phenotyping conducted to study winter wheat varieties under 3 levels of N treatments in the 2020 to 2021 season, followed by automated phenotypic analysis of N response-related traits together with postharvest assessment of N-utilization indices and yield performance. (A) An orthomosaic image acquired from the 2020 to 2021 field experiment that consisted of 486 6-m^2^ winter wheat plots (54 varieties, 3 replicates) under 3 levels of N fertilization treatments (i.e., 0, 180, and 270 kg N·ha^−1^; i.e., N0, N180, and N270). (B) Low-cost drones (e.g., DJI Mavic 2 Pro) and manual measures applied in field phenotyping, which collected wheat varieties performance-related phenotypes across 2020 to 2021 season. (C to E) A series of 2D and 3D imagery generated to represent crop performance at different key growth stages under the 3 N treatments, followed by automated trait analysis using the AirMeasurer platform, which produced a range of plot-based phenotypic traits using spectral, morphological, and textural signals. (F) Correlation analyses between AirMeasurer-derived traits (e.g., canopy coverage and plant height) and manual scoring. (G) Twelve yield-production and N-utilization (Y&N) indices measured during postharvest assessment.

Since 2020, the field experiment was set up in a 2-factor split-plot design, dividing the field into 3 big plot blocks. Each applied with 3 different levels of N treatments (i.e., subplot blocks), including 0, 180, and 270 kg N per hectare (i.e., N0, N180, and N270). Every subplot block consisted of 54 6 m^2^ plots (2 × 3 m), and the entire experiment contained 486 plots with 3 biological replicates (Fig. 1A). The sowing density was kept 2.4 million plants per hectare per season with 25-cm row spacing, suitable for both cultivation and breeding purposes. Soil N, P, and K content was measured before sowing (Table S2). Base fertilizers, 60% of the total N (urea), phosphorus (P_2_O_5_), and potassium (K_2_O) fertilizers, were applied at 0 days after sowing (DAS), followed by 40% N fertilizers at 130 DAS (i.e., 1 day after N fertilization [DAF]), when most of the varieties entered the jointing stage (growth stage [GS] 33). Crops were managed following standard husbandry and agronomic inputs according to local conditions, including fungicides and insecticides applications at GS33 and GS65 (flowering).

### Drone-based field phenotyping, manual scoring, and ground truthing

**Fig. 2.**
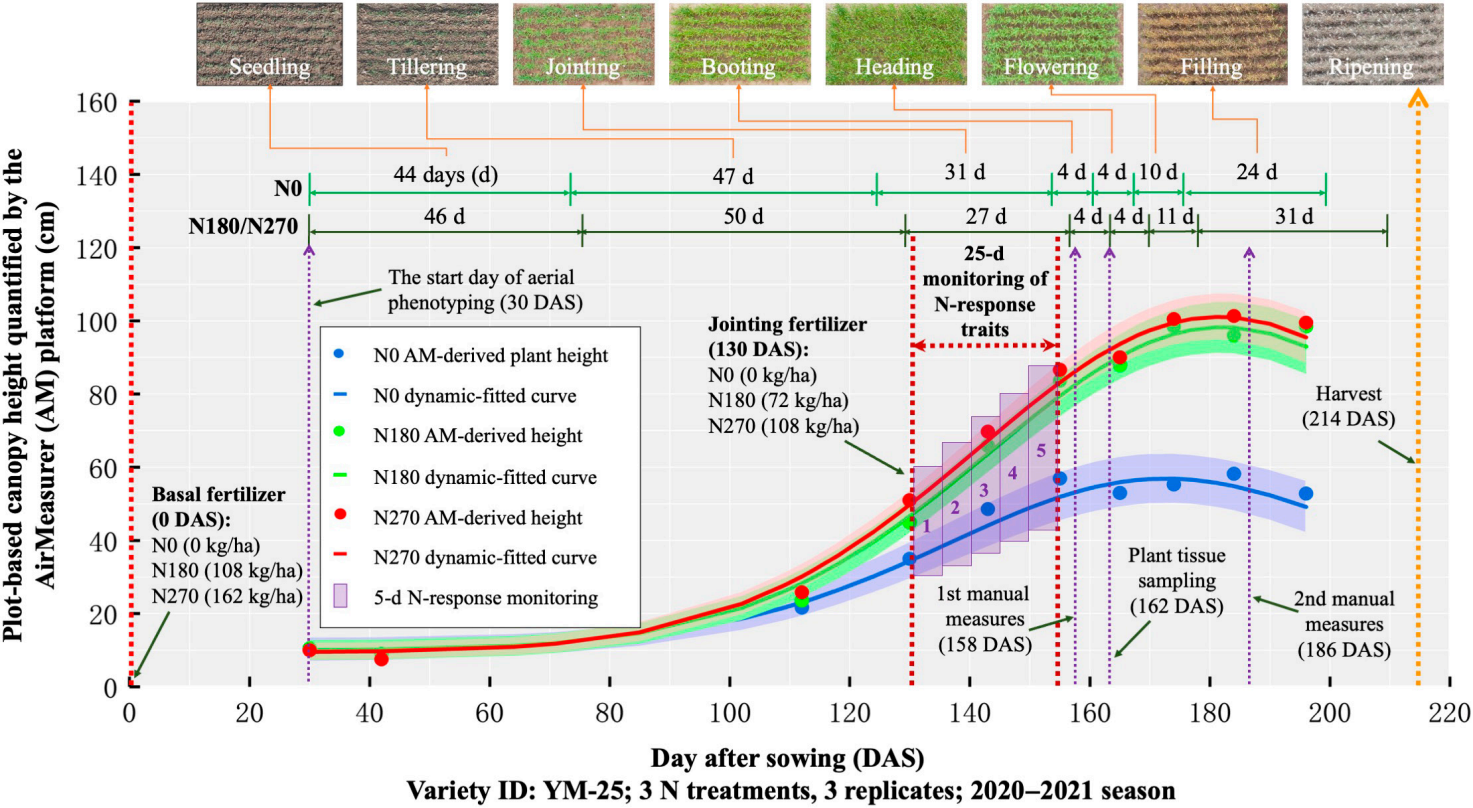
Three height-based N-response profile curves established for a wheat variety (YM-25) trialed in the 2020 to 2021 season under the 3 levels of N treatments (colored blue, green, and red, respectively). Height values (colored dots) were derived by the AirMeasurer platform, and profile curves were produced using the Gaussian fitting function. Plot-based overhead aerial images are presented as references, with colored shading areas denoting 25th- to 75th-percentile confidence intervals. Dates for basal and jointing N fertilizations are denoted with arrows.

We utilized a low-cost drone (Mavic 2 Pro; DJI, Shenzhen, China) to perform low-altitude aerial phenotyping at key growth stages (Fig. 1B, upper). Following recommended practices previously described [45], we installed ground control points and height reference panels in the experiment, followed by the use of DJI GS PRO to perform drone flights with tailored parameters [28] to ensure high-quality aerial imagery acquired at 14- to 15-m altitude. To avoid unnecessary aerial phenotyping, we only performed 10 flights during the season (Fig. 2), from seedling to late grain filling to provide sufficient plant growth and development phenotyping data for phenotypic analysis.

In order to verify computationally derived traits such as plot-based canopy coverage, Fiji/ImageJ [46] was used to manually measure canopy coverage (in pixels; using the autothresholding function) and green leaf index (used to assess vegetation) using plot-level wheat images segmented by the AirMeasurer platform. Besides drone phenotyping, plot-level canopy plant height were also scored manually within a 0.5 × 1 m sampling area in all the 486 plots (Fig. 1B, lower). The manual scoring was normally accomplished 1 to 2 d after the aerial phenotyping to ensure its relevance. Ground truthing was employed to verify the reliability of AirMeasurer-derived static phenotypes, including yield traits such as spike number per unit area and dry biomass of aboveground plants (gram per m^2^ [g·m^−2^]) measured at the heading (GS55), grain filling (GS81), and postharvest stages.

### Phenotypic analysis using the AirMeasurer platform

Drone-collected raw images (over 15 GB per flight) were first preprocessed by the Pix4DMapper software (Pix4D, Lausanne, Switzerland), generating 2D orthomosaics and 3D point clouds containing plot-based morphological, color, and textural signals of plants (Fig. 1C). Then, the AirMeasurer platform [28] was applied to automate trait analysis using the 2D/3D image series and real-time kinematic positioning, producing digital elevation models (i.e., aboveground 3D points), digital surface models (i.e., ground-level 3D points), and a range of plot-level traits (Fig. 1D and E), including morphological traits such as plot-level plant height and canopy coverage, spectral traits such as normalized difference yellowness index (NDYI, for detecting flowering and yellowness to map changes from vegetation to reproduction) and visible atmospherically resistant index (VARI, for assessing canopy-level greenness and vegetation), and textural traits such as 3D Canopy Index (3DCI, for measuring canopy spatial structure) [32] and angular second momenta (ASM, for describing canopy textural uniformity).

### Postharvest N-utilization and yield-performance measurements

We examined N-utilization indices with both physiological and biochemical measurements. First, we randomly sampled 20 wheat plants across a given plot, drying the plants and separating them into stems, leaves, spikes and rachis, and grains. Then, we grouped organ parts and quantified N content using the Automatic Discrete Analyzer Cleverchem 380 (DeChem-Tech, Hamburg, Germany); for measuring key yield components such as grain yield per unit area, spikelet number per spike, thousand grain weight, and dry biomass of aboveground plants (with or without grains), we used the Topu grain test and grain balance instrument (Zhejiang Topu Yunnong Technology, Hangzhou, China; Fig. 1G). Finally, we combined aboveground plant N content, N content without grains, and grain N content with available N in the soil to compute N-utilization indices using formulas reported previously [47]. Twelve Y&N indices were produced, including spike number per unit area, grain number per spike, thousand grain weight, aboveground biomass (g·m^−2^), aboveground straw biomass (g·m^−2^), grain yield (g·m^−2^), N in aboveground biomass (g·kg^−1^), N in straw (g·kg^−1^), grain N content (g·kg^−1^), NUE, NUpE, and NHI.

### PCA for classifying varieties and improving model training

We applied PCA [48] to classify the 54 varieties into 4 N-responsiveness groups using the above 12 Y&N indices. Because many indices were measured, PCA was first used to reduce the dimensionality of the Y&N dataset. Principal components (PCs) were computed based on eigenvalues, contribution rates, and cumulative contribution rates (CCRs) together with weights of all the indices under the 3 N treatments. Then, top 4 PCs, with CCRs greater than 85%, were identified using variance cumulative contributions and component loading coefficients (Note S1), resulting in the N efficiency comprehensive scores (NECS) using the equation below:

$${\text{NECS}}_{i}=\sum_{i=1}^{54}\sum_{j=1}^{4}\lambda_{j}{\mathrm{PC}}_{\mathrm{ij}}$$ (1)

Where *PC_ij_* is the PC value of the *j*th component of the *i*th wheat variety, and 𝜆_𝑗_ is the contribution rate corresponding to the *j*th component.

Similarly, PCA was also utilized when building a supervised ML model called RF-NRES to predict a given variety’s N-responsiveness (i.e., N-dependent yield increase) group. PCA was applied to examine 5-d phenotypic changes (in %) of the 6 target traits during the 25-d period. By computing the variance contribution rate of each PC and their coefficients, PCs with CCRs greater than 85% were selected. Absolute values of loading coefficients were applied to compute weights of the 6 N response-related traits (Note S2), which were then used as hyperparameters when training the RF-NRES model.

### Dynamic phenotypic analysis of N responses

We focused on studying dynamic or temporal features between 1 (the day of N fertilization, i.e., 130 DAS) and 25 DAF through dynamic phenotypic analysis using curve fitting and profile curves. This approach was based on the Gaussian or logistic regression methods reported before [28,49]. We derived profile curves from static phenotypes collected at key growth stages using drone-based phenotyping (Fig. 2). For example, under the N270 treatment, 10 AirMeasurer-derived canopy height values (red-colored dots; Fig. 2) were selected according to key growth stages and used to develop the N270-response profile curve for a variety called “Yangmai 25” (YM-25), representing YM-25’s height changes between seedling (30 DAS) to grain filling (196 DAS) when plant height decreased slightly due to ripening. As the profile curve tended to follow a sigmoid pattern, we chose the Gaussian function [50] to fit N0, N180, and N270 height curves (colored blue, green, and red, respectively; Fig. 2), showing dissimilar height-based response patterns under N treatments. Nevertheless, as some target traits did not follow a sigmoid pattern, we utilized other curve fitting functions [51] such as Fourier transform (Eq. 2; for ASM and NDYI), Gaussian (Eq. 3; for 3DCI and VARI), and Weibull sigmoid fitting (Eq. 4; for canopy coverage) to produce curves. The evaluation and selection of fitting functions for the traits are listed in Table S3. The equations used to produce N-response curves are as follows:

$$\text{Fourier}\left(x\right)=\sum_{n=1}^{u}\left(a_{n}\text{cos}\frac{\mathrm{n\pi x}}{L}+b_{n}\text{sin}\frac{\mathrm{n\pi x}}{L}\right)$$ (2)

$$\text{Gaussian}\left(x\right)=a\times e^{\frac{-{\left(x-b\right)}^{2}}{2c^{2}}}$$ (3)

$$\text{Weibull}\left(x\right)=L\times \left(1-e^{{\left(-\left(\frac{x}{x_{0}}\right)\right)}^{b}}\right)$$ (4)

For Fourier, *x* denotes the number of DAS, *u* equals to 6 key growth stages (i.e., from seedling to grain filling), *a_n_* and *b_n_* stand for change rates of the Fourier curve at the 6 stages; for Gaussian, *x* denotes DAS, *a* represents the maximum canopy height, *b* is the value on x-axis when the Gaussian curve reaches peak, and *c* is the standard deviation of the curve; for Weibull, *x* denotes DAS, *L* is the maximum value of the Weibull curve, *b* is the growth rate of the curve, and *x*_0_ is the number of DAS when the curve reaches its peak.

We also developed an algorithm to extract subcurves from the profile curves to study N response. First, we located the 130- to 154-DAS region (i.e., 1 to 25 DAF on x-axis; Fig. 2) based on the 25-d N-response phase reported previously [52,53]. Then, we focused on dividing the region into five 5-d sections (purple shading rectangles, i.e., 1 to 5, 6 to 10, 11 to 15, 16 to 20, and 21 to 25 DAF). Finally, we computed height changes (y-axis) for every section using the compound growth rate (CGR) equation [54] due to CGR’s advantage in considering compounding effect of phenotypic changes (i.e., unaffected by different volatility levels across target traits during N response):

$${\mathrm{CGR}}_{\text{height}}={\left(\frac{f{\left(x_{\left(i+4\right)}\right)}_{\text{height}}}{f{\left(x_{i}\right)}_{\text{height}}}\right)}^{\frac{1}{5}}-1$$ (5)

where *f*(*x_i_*)*_height_* denotes the Gaussian-fitted height curve, and *i* is the beginning day of a 5-d N-response period, which equals to 1, 6, 11, 16, or 21 DAF.

### ML-based predictive modeling

To identify varieties with better N responsiveness phenotypes, we first employed a range of supervised ML models such as support vector machines, Bayesian classifier, K-nearest neighbor, Adaptive Boosting (AdaBoost), eXtreme Gradient Boosting (XGBoost), and random forest (RF) [55]. After comparing these ML models, we chose the RF-based model and applied PCA-derived weights as hyperparameters to fine-tune the prediction model (see Note S3), resulting in a model called RF-NRES that yielded the best predictive power to classify N-responsiveness groups.

### Correlation analysis to verify AirMeasurer-derived traits

The square of the correlation coefficient (*R*^2^) and Root-mean-square errors were used to assess linear regression between manually scored and AirMeasurer-derived static traits such as plot-based canopy height, canopy coverage, and canopy-level leaf vegetation (Fig. 1F and Fig. S1). Also, correlation analyses were conducted between dynamic phenotypes (e.g., growth rates) derived from static traits based on the profile curves, validating AirMeasurer-derived phenotypes and selecting suitable fitting functions (Table S3).

### GWAS analysis

To explore the biological relevance of dynamic phenotyping in wheat genetic mapping, we genotyped the 54 wheat varieties using the 55K microarray data [56,57]. We used PLINK (v1.9) to perform quality control of genotype data [58], Structure (v2.3.4) to analyze the population structure [59], as well as the general linear model in Tassel (v5.2) to perform genome-wide association study (GWAS) analysis [60] to identify the associated-loci controlling static and dynamic phenotypes during N response, with the population structure as covariates. Markers with missing data (>10%) or low minimum allele frequency (<5%) were removed. Single-nucleotide polymorphisms (SNPs) with a *P* value < 1e-5 and across 2 seasons were retained and then referenced to the Chinese spring reference genome (IWGSC Ref Seq v1.0) and the genome annotation file, Ref Seq Annotation v1.1 [61]. Finally, we used Bedtools (v2.18) to obtain potential candidate genes from significant SNPs, within up- and down-stream 500-kb regions [62]. The CMplot package in R scripting language [63] was employed to draw the SNP density map, Manhattan and quantile-quantile plots. Linkage disequilibrium and haplotype analysis were performed using Haploview [64]. It is worth noting that the GWAS analysis performed here aimed to explore a new approach to utilize N response-related phenotypic changes in genetic mapping. We did not intend to discover novel SNPs nor to validate SNPs identified.

## Results

### The classification of 4 N-responsiveness groups

To identify varieties’ performance in terms of yield production and N utilization (i.e., N responsiveness), we first computed the 2-season 12 Y&N indices of the 54 varieties (Data S1) under N0, N180, and N270 treatments. Statistical analysis of the indices are provided in Table S4 and Fig. S2. Then, we used PCA to compute eigenvalues, contribution rates, CCRs, and weights of PCs of these indices, followed by the selection of components with their CCRs greater than 85%. The results (Tables S5 to S7) indicated that: (a) under N0, grain N content, NUE, vegetative organ biomass, and aboveground biomass had greater weights in PCA (9.3% to 9.8%); (b) under N180, grain N content, NUE, aboveground biomass, and grain yield had greater weights (9.0% to 9.4%); (c) under N270, vegetative organ N content, grain yield, and NHI had greater weights (9.0% to 9.4%). Building on the variance contribution rates, NECS values under 3 N treatments were calculated using the equations below:

$${\text{NECS}}_{N0}=33.6\%{\mathrm{PC}}_{1}+23.6\%{\mathrm{PC}}_{2}+18.0\%{\mathrm{PC}}_{3}+12.4\%{\mathrm{PC}}_{4}$$ (6)

$${\text{NECS}}_{N180}=35.2\%{\mathrm{PC}}_{1}+23.7\%{\mathrm{PC}}_{2}+16.5\%{\mathrm{PC}}_{3}+11.9\%{\mathrm{PC}}_{4}$$ (7)

$${\text{NECS}}_{N270}=40.6\%{\mathrm{PC}}_{1}+20.8\%{\mathrm{PC}}_{2}+18.6\%{\mathrm{PC}}_{3}+10.3\%{\mathrm{PC}}_{4}$$ (8)

where *PC*_1−4_ are top PCs computed by PCA, and coefficients of PCs are contribution rates.

**Fig. 3.**
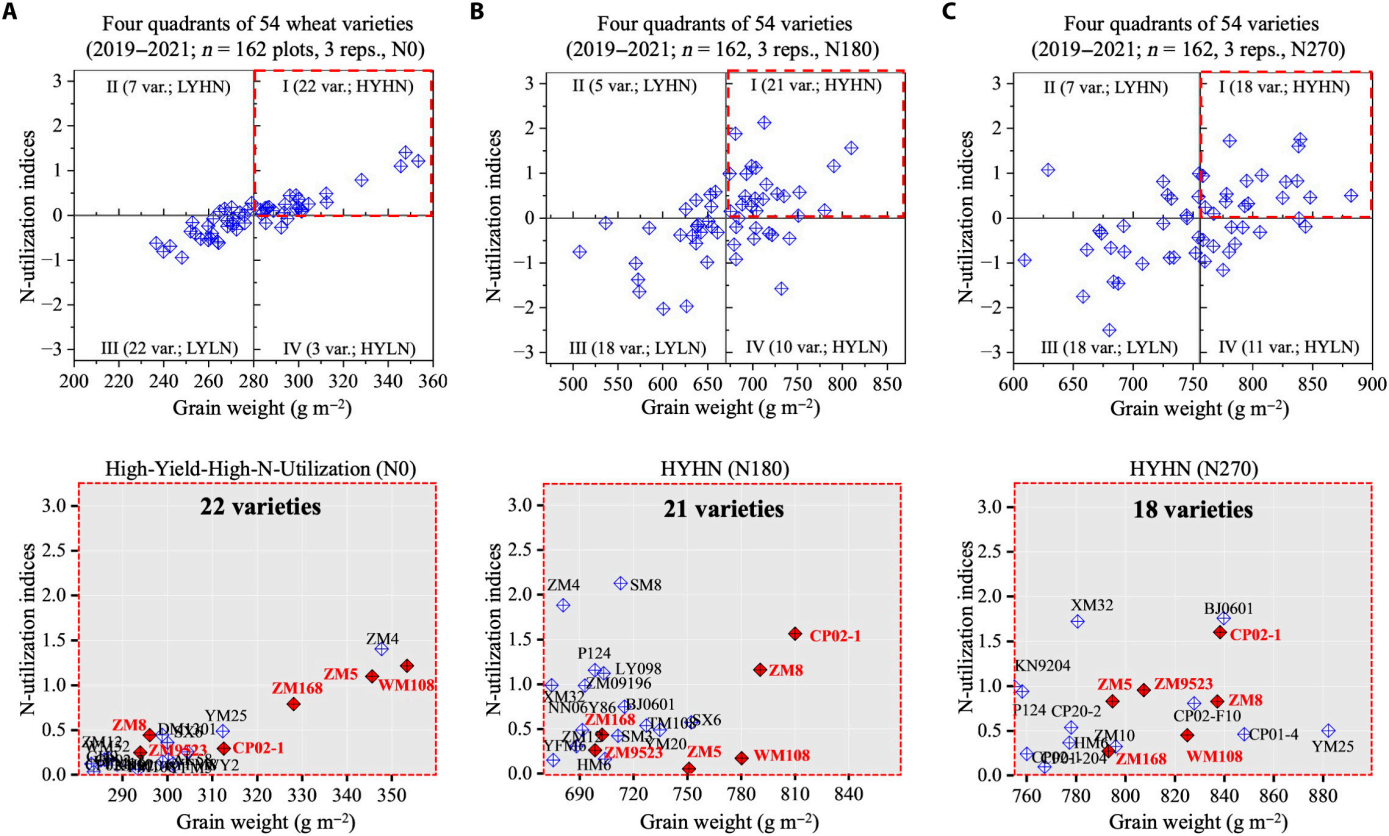
Three 4-quadrant models (upper) categorize 54 wheat varieties into 4 N-responsiveness groups based on yield-performance and N-utilization indices under N0, N180, and N270 applications and across the 2019 to 2021 seasons. (A to C) Four groups of 54 wheat varieties identified (upper) based on their Y&N indices across the 2019 and 2021 seasons. The HYHN group (lower) was detailed, and wheat varieties consistently performed well in N responsiveness were highlighted with red rhombuses.

We ranked the 54 varieties (Table S1) under 3 N applications using NECS values (Tables S8 to S10). Then, according to the average value of NECS, varieties were classified into 4 N-responsiveness groups, i.e., High-Yield-High-N-Utilization (HYHN, Class I), Low-Yield-High-N-Utilization (LYHN; Class II), Low-Yield-Low-N-Utilization (LYLN; Class III), and High-Yield-Low-N-Utilization (HYLN, Class IV). Because most of the varieties belonged to Class I and III (Fig. 3), we therefore only listed HYHN and LYLN varieties in Table.

**Table.** HYHN (Class I) and LYLN (Class III) wheat varieties identified using NECS measures

N treatments	HYHN varieties	LYLN varieties
N0	CP02-F10, CP02-1, CP02-2, AK58, DM1301, GH9, KN9204, PM108, SX6, WY2, WM108, WM52, YFM5, YFM8, YM16, YM25, ZM9523, ZM12, ZM168, ZM4, ZM5, ZM8	CP01-3, CP01-204, CP01-4, CP02-6-1, CP20-1, P14, HM30, LY098, NN06Y86, NM13, NM26, NMZ1019, NZM1, SM3, SM8, SM553, WL12, XM32, YFM6, YM158, YM20, ZM11
N180	CP02-1, P124, BJ0601, HM6, LY098, NN06Y86, SX6, SM3, SM8, TM108, WM108, XM32, YFM6, YM20, ZM9523, ZM09196, ZM12, ZM168, ZM4, ZM5, ZM8	CP01-3, CP01-4, CP02-21, CP02-6-1, CP03-1, CP20-1, AK58, HM30, NM13, NMZ1019, NZM1, SKM1, SM553, WM52, WL1216, YFM5, YM158, ZM10
N270	CP01-204, CP01-4, CP02-F10, CP02-1, CP02-6-1, CP20-2, P124, BJ0601, HM6, KN9204, WM108, XM32, YM25, ZM9523, ZM10, ZM168, ZM5, ZM8	CP01-3, AK58, HM30, LY098, NN06Y86, NM26, NMZ1019, PM108, SKM1, SM553, WY2, WM52, WL1216, YFM6, YFM8, ZM09196, ZM11, ZM4
Overlapped varieties	CP02-1, WM108, ZM9523, ZM168, ZM5, ZM8	CP01-3, HM30, NMZ1019, SM553, WL1216

To visualize the identified varieties and their N-responsiveness groups, 3 scatter plots were produced (Fig. 3, upper), showing the 4 groups and their associated varieties under N0, N180, and N270 treatments. Among them, we noticed that 6 wheat varieties (i.e., ZM5, ZM8, ZM9523, ZM168, CP02-1, and WM108, colored red in Fig. 3) consistently performed well in terms of Y&N, indicating better N responsiveness across the 3 N treatments.

### Static phenotypes of N-response traits using drone phenotyping at 10 time points

After classifying the varieties into 4 N-responsiveness groups, we used the AirMeasurer platform to perform phenotypic analysis of the 6 N-response traits (i.e., plant height, canopy coverage, NDYI, VARI, 3DCI, and ASM) using spatial, spectral, and textural signals collected by drone phenotyping. The aerial phenotyping was performed on 486 plots at 10 time points between 2019 and 2021 seasons, covering key growth stages from 30 DAS (seedling, GS15) to 196 DAS (grain filling, GS85). Based on static phenotypes, line charts of the 6 traits were produced (Fig. S3), demonstrating changing profiles of the traits during the season. Phenotypic analysis across 2019 to 2021 seasons is also provided (Data S2), including information such as N treatments, plot location, and replicates. To facilitate researchers to reproduce our work, we uploaded testing aerial images, trait analysis, and Jupyter notebooks to GitHub repository (https://github.com/The-Zhou-Lab/Nitrogen-response-traits/releases).

### Dynamic phenotypes of N-response traits using curve fitting

**Fig. 4.**
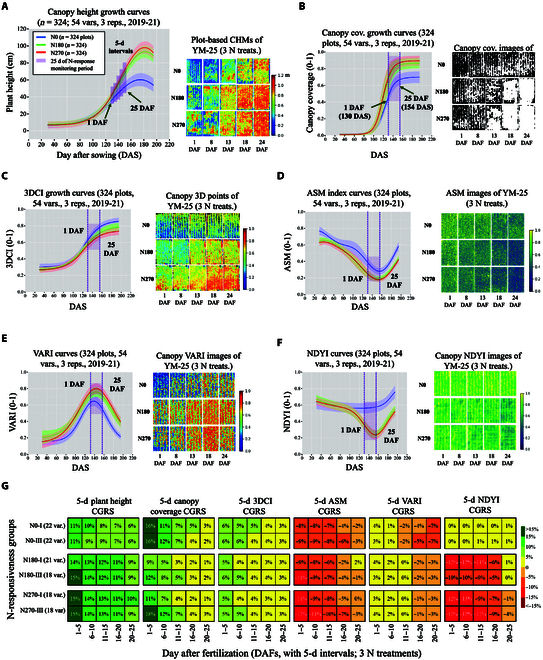
Growth curves of 6 target traits generated using dynamic phenotyping algorithms for 54 wheat varieties, including plant height, canopy coverage, 3DCI, ASM, VARI, and NDYI; subcurves (highlighted by light purple shading areas) extracted between 1 and 25 DAF to represent phenotypic changes caused by different N treatments since the jointing fertilization (i.e., 1 DAF); plot-based overhead visual representations of the 6 traits provided as references; a CGR performance matrix created to provide quantifiable phenotypic changes of the 6 traits with 5-d intervals. (A) The AirMeasurer-derived plant height growth profiles under N0, N180, and N270 treatments (colored blue, green, and red; left); each curve was produced using static phenotypes measures at 10 time points from 324 plots across 2019 to 2021 seasons. The purple vertical shading lines represent the 25-d monitoring period (i.e., 1 to 25 DAF); 5-d intervals were used to dissect phenotypic changes of wheat plants’ responses to N treatments. Colored shading areas denote 15th- to 85th-percentile confidence intervals. Pseudo colors applied to the plot-level CHMs (right) according to a unified height scale bar (0 to 1.2 m). (B to F) AirMeasurer-derived curves for the other target traits (i.e., canopy coverage, 3DCI, ASM, VARI, and NDYI). (G) The CGR matrix listing a comprehensive overview of 5-d phenotypic changes of 6 selected traits after jointing N fertilization. The traits were quantified from 54 wheat varieties (3 replicates per N treatment; 2 seasons) and dynamic phenotypic analysis were applied to derive changes rates. In the matrix, the varieties were grouped into 4 N-responsiveness groups. Pseudo color applied to highlight the CGRs of the traits according to a scale bar (<−15% to >15%; right).

The accuracy of drone-based phenotyping could be impacted by complex field conditions such as nature illuminance, rainfall, and high or gusty wind [65]. Without calibration, this often led to problems in phenotypic analysis (e.g., the profile curves in Fig. S3 contain variability). Hence, we utilized the 10 static phenotypes to establish profile curves using curve fitting functions such as Gaussian, Fourier, and Weibull. Using the fitted curves (Fig. 4A to F and Table S3), not only could we identify changing profiles of the 6 traits across the season with outliers removed, but we also examined phenotypic changes during N response (i.e., 130 to 154 DAS; light purple shading areas; Fig. 4A to F), under N0, N180, and N270 treatments (colored blue, light green, and red, respectively).

For an example trait, canopy height, the Gaussian-fitted height curves (Fig. 4A) show that the height growth accelerated after the jointing fertilization (1 DAF) under N180 (40 to 75 cm on average) and N270 (40 to 80 cm) compared to N0 (35 to 50 cm), suggesting height differences enlarged by the N treatments (Fig. 4A, left). To visualize the phenotypic changes, we retrieved the canopy height models (CHMs; Fig. 4A, right; pseudo colors applied according to a height scale bar, 0.0 to 1.2 m) generated by using the drone-acquired 3D point clouds at 130, 138, 143, 148, and 154 DAS (i.e., 1, 9, 14, 19, and 25 DAF). The pseudo-colored CHMs indicated that height differences under the 3 N treatments diverged at 11 to 15 DAF considerably, which complied with what was observed in the field.

Similarly, for canopy coverage (Fig. 4B, left), both N180/N270 curves performed similarly, reaching 90% plot-level coverage at 25 DAF, when N0 profile curve plateaued around 70%. The effects of N applications deviated phenotypic changes of canopy coverage at 11 to 15 DAF, which could be seen using canopy masks (Fig. 4B, right). 3DCI (Fig. 4C, left) divided the 3 N treatments into 3 groups based on canopy structural variance (Fig. 4C, right) that became either uniform (e.g., N270) or sparse (e.g., N0). ASM was employed to quantify plant canopy density (Fig. 4D, left), indicating that all the varieties achieved the maximum density at 25 DAF (Fig. 4D, right). We also produced profile curves for spectral traits such as VARI (Fig. 4E) and NDYI (Fig. 4F). N180/N270 VARI values were 10% to 15% higher than those under N0, suggesting greener canopies. The N0 VARI curves peaked at 6 to 10 DAF with N180/N270 VARI curves peaked at 11 to 15 DAF, indicating different dates for booting (GS41 to GS49). N180/N270 NDYI curves dropped gradually during 1 to 25 DAF, whereas N0 curve maintained its tendency, suggesting that N180/N270 treatments led to an elongated vegetation.

### The dissection of the N response-related phenotypic changes

Using the subcurves extracted between 1 and 25 DAF, we could further dissect and quantify phenotypic changes of the 6 target traits during N response. First, we extracted subcurves from the profile curves between 1 and 25 DAF, followed by dividing the curves into five 5-d sections (i.e., 1 to 5, 6 to 10, 11 to 15, 16 to 20, and 21 to 25 DAF). Then, 5-d CGRs were computed, representing morphological (e.g., height and canopy coverage), textural (3DCI and ASM), and spectral (VARI and NDYI) changes. Because we chose to compare distinct differences between HYHN (Class I) and LYLN (Class III) varieties in order to identify desired N-response phenotypes for better N responsiveness, we created a CGR performance matrix (Fig. 4G) with each cell showing phenotypic changes at 5-d intervals. Pseudo colors were also applied according to a unified scale bar, from dark green (i.e., CGR > 15%) to dark red (i.e., CGR < −15 %). We detailed daily CGR values of the 6 N response-related traits in Data S3, and a complete CGR matrix (including HYLN and LYHN groups) is presented in Fig. S4.

Using the matrix and the mean values of CGRs at 5-d intervals, key phases for phenotypic changes identified for HYHN varieties were: (a) height (N0, 1 to 10 DAF; N180, 1 to 20 DAF; N270, 1 to 25 DAF), (b) canopy coverage (N0, 1 to 15 DAF; N180/N270, 1 to 10 DAF), (c) 3DCI (no major changes), (d) ASM (N0/N180/N270, 1 to 15 DAF), (e) VARI (N0/N180/N270, 1 to 10 DAF), and (f) NDYI (N0, no major changes; N180/N270, 1 to 15 DAF). Similarly, key phases for LYLN varieties were: (a) height (N0, 1 to 10 DAF; N180/N270, 1 to 20 DAF), (b) canopy coverage (N0/N180/N270, 1 to 15 DAF), (c) 3DCI (no major changes), (d) ASM (N0, 1 to 20 DAF; N180, 1 to 15 DAF; N270, 1 to 20 DAF), (e) VARI (N0/N180/N270, 1 to 10 DAF), and (f) NDYI (N0, no major changes; N180/N270, 1 to 20 DAF). In general, under N0 treatment, HYHN varieties had a steadier developmental pace than LYLN varieties; under N180, HYHN varieties’ canopy coverage developed slower than those in the LYLN group, and HYHN varieties’ NDYI values noticeably decreased faster; under N270, HYHN varieties’ canopy coverage, ASM, and NDYI values changed slower than LYLN varieties, suggesting an elongated vegetation growth. Under N180 and N270 treatments, HYHN and LYLN varieties performed similarly, except HYHN varieties showed longer and steadier phenotypic changes, indicating that N fertilizers had a longer and more stable effects on these varieties.

### Using N-response phenotypes to model N responsiveness

**Fig. 5.**
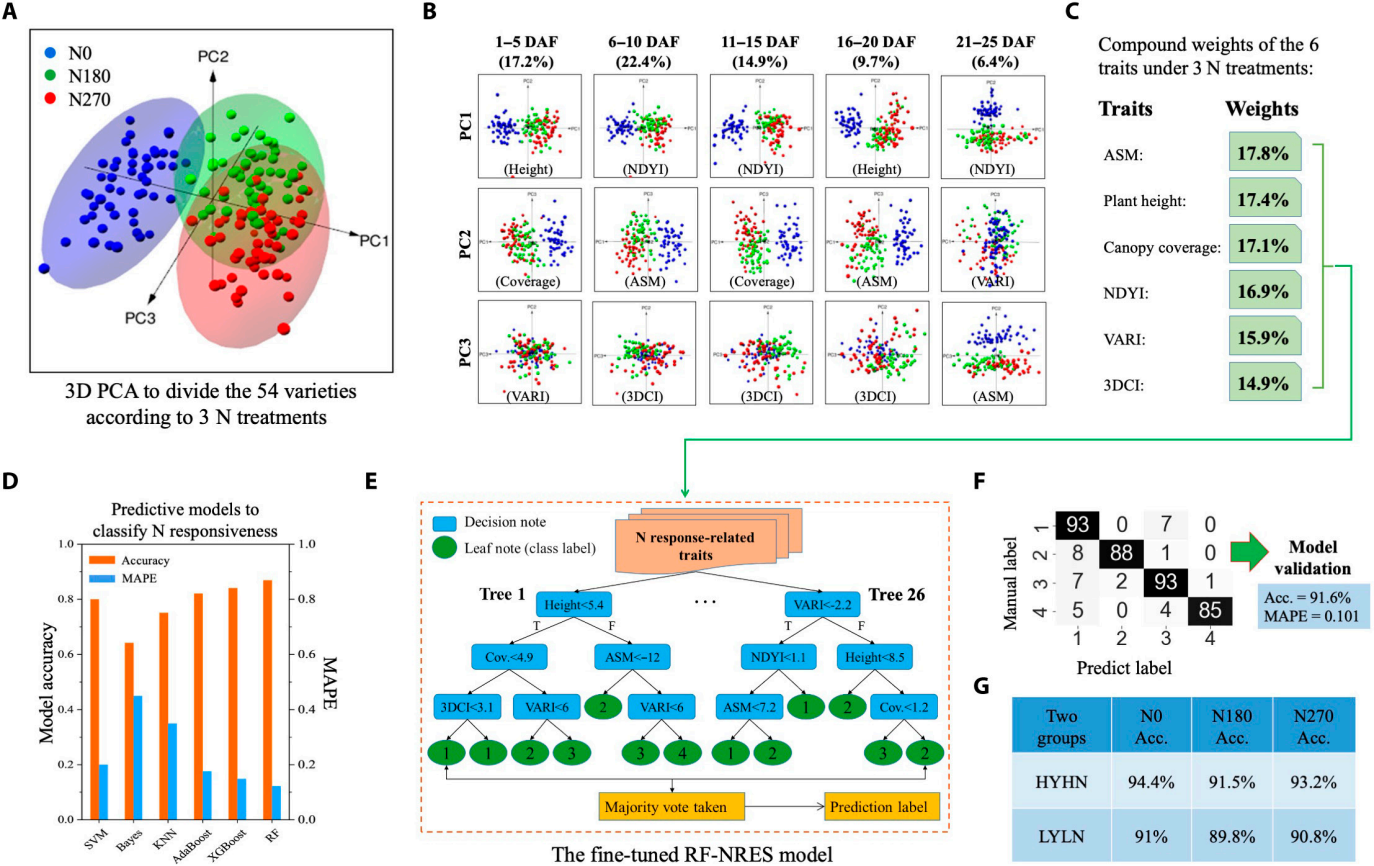
An ML-based model called RF-NRES established to classify N-responsiveness groups based on N response-related traits, phenotypic changes at 5-d intervals, and compound weights computed by applying PCA after jointing fertilization. (A to C) 3D PCA performed to study whether varieties could be divided according to the 3 N treatments, followed by computing weights of the 3 most relevant traits (i.e., with CCRs greater than 85%) with 5-d intervals. Identified traits and their weights were used to model N-responsiveness groups in wheat. (D) A range of supervised ML techniques employed to seek a suitable learning model to forecast N-responsiveness groups based on N-response phenotypes of the 6 traits as inputs. (E and F) An RF-based model called RF-NRES established that yielded the best accuracy in predicting N-responsiveness varieties (91.6% across the 4 groups), with mean absolute percentage error (MAPE) reported.

The above approach not only provided us with a new method to quantify N response-related trait, but it also supplied the dissection of phenotypic changes at key N-response phases, which enabled us to gain an insight into phenotypic variation caused by N applications. In fact, researchers [66–68] already suggested that performance- and yield-related traits and their phenotypic information could be used to identify the performance of NUE and N responsiveness in wheat. Hence, with an aim of pinpoint N responsiveness using N response-related phenotypes, we first applied PCA to study the 6 target traits and their phenotypic changes during 1 to 25 DAF (Fig. 5A), which verified that the 54 varieties could be largely separated according to the 3 N treatments. Then, we located top 3 PCs with CCRs greater than 85%, followed by the identification of key N response-related traits based on trait-based weights (Fig. 5B and Table S11; see Note S2 for detailed weight calculation) at 5-d intervals. This suggested that the jointing fertilization (i.e., N180/N270) had relatively high impacts on the N response-related traits during 1 to 5 DAF (17.2%), peaked in 6 to 10 DAF (22.4%), and gradually decreased from 11 to 15 DAF (14.9%) onwards, largely corresponding to key N-response phases identified in the last section (Fig. 5B). Finally, weights of the traits were combined into compound weights of the 6 traits (Fig. 5C).

Utilizing the compound weights, we trialed a range of supervised ML models such as support vector machines, Bayesian, K-nearest neighbor, AdaBoost, XGBoost, and RF to model the relationship between N-response phenotypes as input parameters and N-responsiveness groups as targets. We followed the recommended model training procedure [69], dividing the data (*n* = 810) into training (70%; *n* = 774, after data augmentation to balance different N-responsiveness groups), testing (20%; *n* = 427, after augmentation), and verification (10%; *n* = 387, after augmentation) sets (Data S4 and S5). After comparing the classification results of the ML models (Fig. 5D), the RF classifier yielded the best accuracy (86.4%). After that, we chose the RF-based model and fine-tuned its hyperparameters by incorporating the compound weights into the model training (Fig. 5E), with output labels corresponded with the 4 N-responsiveness groups. The final model (with 26 trees and a maximum depth of 16) is called RF-NRES, which achieved an accuracy of 91.6%, 5.2% above the standard RF classifier (mean absolute percentage error = 0.101) when predicting N-responsiveness groups for the varieties. We also conducted a 5-fold cross-validation to verify the accuracy of the model (Fig. 5F), followed by the use of out-of-bag testing set, resulting in average classification accuracies of 93.0% for HYHN varieties and 90.5% for LYLN varieties across 3 N treatments (Fig. 5G).

### The use of static and dynamic phenotypes in GWAS

**Fig. 6.**
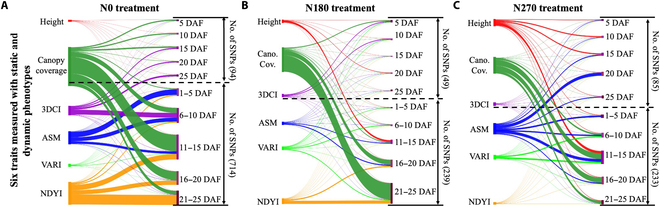
Sankey plots used to represent the association between static and dynamic phenotypes of the 6 N response-related traits (colored differently) and significant SNPs identified using GWAS analysis during N response across 2019 to 2021 seasons. The width of data flows correlates with the SNP number. (A to C) Sankey plots used to present static and dynamic traits (for the 6 target traits) associated with the number of significant SNPs (i.e., above the significance threshold set in GWAS analysis), with the width of data flows proportional to the number of SNPs, showing the use of static and dynamic traits in dissecting genetics when wheat plants responded to different N treatments.

Besides using static and dynamic phenotypes to study N responsiveness and N-response phenotypes, we further explored the use of dynamic phenotyping to associate genetics underlying N responsiveness. GWAS was performed using the Wheat55K SNP Array [57,70]. After removing low-quality markers, 44,215 SNP markers were obtained, covering 13,531.8 Mb (Table S12 and Fig. S5A). The population structure suggested that the examined varieties could be divided into 3 subgroups (Fig. S5B). We compared genetic mapping results generated by using both static and dynamic phenotypes such as change rates of plant height between 1 to 5, 6 to 10, 11 to 15, 16 to 20, and 21 to 25 DAF (i.e., *Height*_1−5_, *Height*_6−10_, *Height*_11−15_, *Height*_16−20_, and *Height*_21−25_; the below part on the secondary y-axis to the right in Fig. 6A), as well as height measured at 5, 10, 15, 20, and 25 DAF (i.e., *Height*_5_, *Height*_10_, *Height*_15_, *Height*_20_, and *Height*_25_; the upper part on the secondary y-axis in Fig. 6A). Clearly, dynamic phenotypes associated with more significant SNPs compared with those using static phenotypes. For example, under N0 (without jointing fertilizers and hence most of the N0-varieties approached flowering, GS61, at 25 DAF), 94 SNPs were associated with static phenotypes across all the 6 traits, whereas 714 were associated with dynamic phenotypes; similarly, under N180 and 270 treatments (most of the varieties approached heading, GS51, at 25 DAF), static phenotypes connected with 49 and 85 SNPs compared with dynamic phenotypes associated with 239 and 233 SNPs, respectively (Data S6 and S7).

SNPs identified using 5-d dynamic phenotypes during N response were also varied. Under N0 treatment, *CanopyCoverage*_6−20_ associated with 287 SNPs compared with only 27 SNPs identified using static traits such as *CanopyCoverage*_10,15,20_; the number of identified SNPs (*n* = 157) peaked at 11 to 15 DAF, whereas only 13 SNPs identified using *CanopyCoverage*_15_. There were 45, 52, and 185 SNPs associated with 3*DCI*_6−10_, *ASM*_1−15_, and *NDYI*_11−25_, respectively (Fig. 6A). Under N180 treatment, SNPs identified were primarily associated with dynamic phenotypes (Fig. 6B), including *Height*_11−15_ (7 SNPs), *CanopyCoverage*_16−25_ (150 SNPs), *ASM*_11−20_ (12 SNPs), *VARI*_1−10_ (8 SNPs), and *NDYI*_16−25_ (30 SNPs). Under N270, dynamic phenotypes such as *Height*_11−15_ (21 SNPs), *CanopyCoverage*_11−20_ (82 SNPs), *ASM*_1−15_ (41 SNPs), and *VARI*_6−15_ (22 SNPs) associated with more SNPs compared with static phenotypes at time points such as 5, 10, 15, 20, and 25 DAF (Fig. 6C).

The above GWAS analysis suggests that greater phenotypic changes during N response normally led to considerably more significant SNPs. Noticeably, static phenotypes such as *Height*_20_ (3 SNPs, under N180), *Heigth*_5,10,15_ (16 SNPs, under N270), 3*DCI*_5,10_ (10 SNPs, under N180), and *ASM*_15,20,25_ (35 SNPs, under N270) have associated with more SNPs than those identified at other time points, which correlated with phases that had the most active N-response changes. Besides computationally derived phenotypes, we also used plant height and canopy coverage traits manually scored at 28 DAF to conduct GWAS analysis, which did not lead to any significant SNP (Fig. S5C and D). The above GWAS results are promising to improve the present approach that we conduct genetic mapping. However, due to the small population studied in the field experiment, it is important to note that our intention was not to validate the identified SNPs nor to discover new SNPs. Therefore, we chose to further elaborate the GWAS results in Discussion.

## Discussion

As one of the most important nutrients, N availability could impact substantially on the plant’s yield production and grain quality [71]. Depending on varieties, growth stages, N supply, and environmental conditions, N uptake, assimilation, and utilization in wheat can vary greatly, leading to the use of field phenotypes to assess dissimilar N responsiveness [13]. Nevertheless, many phenotyping methods relied on taking snapshots of N response-related traits or proxies at specific time points, which missed its dynamic nature. This study focused on establishing methods to measure temporal changes of 6 target traits using morphological, spectral, and textural signals acquired by drone phenotyping. We produced profile curves of the traits using fitting functions to represent phenotypic changes during N response, which enabled us to dissect N-response phenotypic changes. Hence, in-depth analysis could be achieved to compare phenotypic differences between varieties, facilitating us to classify N-responsiveness groups with increased confidence. Moreover, we utilized the 5-d phenotypic changes to explore underlying genetics of N responsiveness using GWAS, resulting in some genetic loci relevant to N utilization even with a small population. This indicates the biological relevance of our work.

### Dynamic phenotypic analysis applied to study dynamic phenotypes

Recent advances in plant phenotyping have enabled the collection of time series-based datasets that facilitate researchers and breeders to study traits in a temporal manner [28]. While a large number of images and sensor data have been collected, how to extract meaningful information from the big data is still challenging [72]. In our case, we computed static phenotypes of 6 target traits at key growth stages as data points. Then, we developed evaluation metrics (Table S3) to choose suitable curve fitting functions according to the data points, resulting in profile curves to represent changing profiles of the traits during the season. To validate the produced curves, we performed correlation analysis using manually scored plot-level plant height and canopy coverage, followed by the comparison of profile curves produced in the 2019 to 2020 and 2020 to 2021 seasons (Fig. S6). Utilizing the fitted curves, we were able to: (a) identify and remove outliers caused by phenotyping errors during the season; (b) quantify phenotypic changes during N response (i.e., 1 to 25 DAF), so that detailed dynamic features and change patterns caused by N applications could be characterized; and (c) dissect N response phenotypes even further, measuring 5-d temporal changes of target traits and thus pinpointing key N-response phases under different N treatments through the CGR matrix (Fig. 4G). To the best of our knowledge, this approach demonstrates novel methodological advances in dissecting and quantifying phenotypic changes to study N-response dynamics in wheat.

### The classification of N responsiveness and modeling

Much research has indicated the importance of the timing and amount of N fertilizations as well as their connections with N responsiveness [73]. In our case, we applied PCA to examine 12 manually scored Y&N indices, which facilitated us to establish the NECS measures (Eq. 1) that could be applied to classify wheat varieties into 4 N-responsiveness groups (i.e., HYHN, LYHN, LYLN, and HYLN). This provided the foundation for us to associate key N-response phases and phenotypic changes listed in the CGR matrix with N responsiveness in wheat, so that both yield production and N utilization could be combined to select desired phenotypes and varietal responses during N response. Additionally, we also used the dissected phenotypes in ML modeling. Based on the 4 N-responsiveness groups, we incorporated PCA-derived compound weights of the 6 target traits into the model establishment, resulting in the RF-NRES ensemble model that used N-response phenotypes to predict yield performance and N utilization (i.e., N responsiveness) with high accuracies (Fig. 5F and G). Also, as a RF-based model, the RF-NRES explored nonlinear features among input parameters, which could be valuable when studying complicated relationships between genotypes, treatments, and environmental factors (e.g., G×E×M) under complex field conditions.

### Dynamic phenotypes in genetic mapping

In human pathology, intermediate phenotypes and phenotypic changes were used to seek disease-associated genetic variants even with a small population as phenotypic changes positioned between genetic variation and diseases could increase the mapping power in finding disease mechanisms [74]. Similarly, in plant research, intermediate phenotypes has also been employed to derive biomarkers for genotypic N response [34]. In fact, spatial and temporal phenotypic variations associated with plant’s responses to external stimuli could increase the statistical power of association studies when studying expression patterns of target genes and their regulators [75].

In our case, we first assessed whether dynamic phenotyping could be used as a suitable approach to enable genetic association for N response-related traits. This led to more significant SNPs identified using dynamic phenotypes compared with those using static phenotypes (Table S13). Because a larger number of SNPs was likely to provide a more comprehensive view of genetic variation [76], we trust that dynamic phenotypes are likely to help us capture a broader range of genetic differences between genotypes and hence the increase of statistical power to fine-map trait-associated genomics. Still, the quality of SNPs (e.g., genotyping errors) and population diversity (i.e., the relevance) also should be considered [77]. Hence, we also verified the biological relevance of the identified SNPs.

**Fig. 7.**
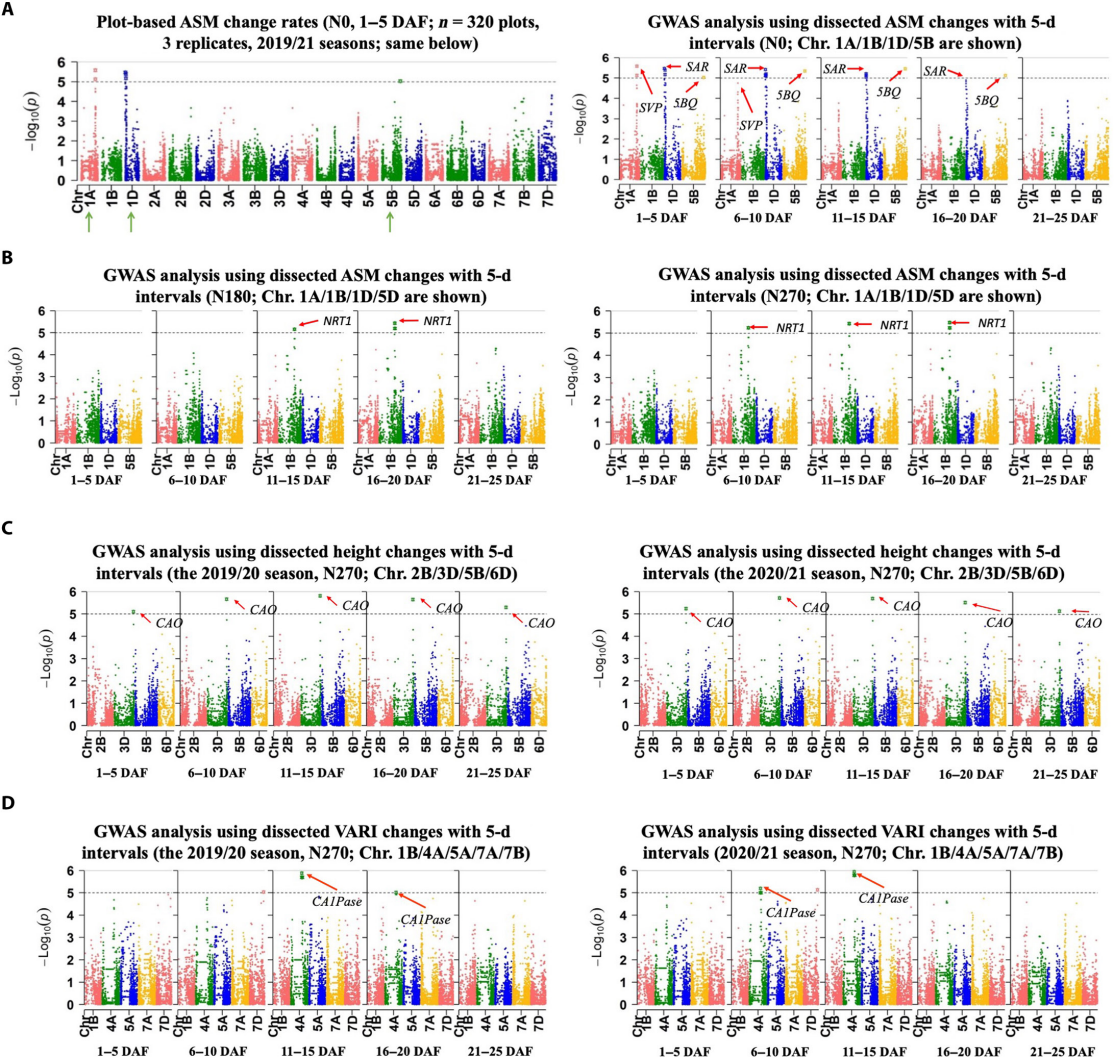
SNPs identified using phenotypic changes through GWAS, producing genetic loci and significant levels during 5-d phases under 3 N treatments. Traits used are ASM (canopy-level texture), height (plant height), and VARI (canopy-level plant greenness and vegetation index). The significance threshold is shown by horizontal gray dotted lines. Known genes that colocate with significant genetic loci are indicated by red arrows. (A and B) Using the ASM trait, the strongest signals on chromosome (*Chr*.) 1A, 1D, and 5B were close to the *SVP*, *SAR*, and *5BQ* genes under the N0 treatment, whereas a strong signal on *Chr*. 1B close to the *NRT1* gene identified under the N180 and N270 treatments during 6 to 20 DAF. The 5-d GWAS demonstrates different loci and significant levels that contributed to the regulation of ASM (i.e., canopy uniformity) under the 3 N treatments. (C and D) Manhattan plots produced to compare dynamic phenotypes for traits such as changing rates of height and VARI. The SNP identified on *Chr*. 3D was close to the *CAO* gene during 1 to 25 DAF, and the SNP identified on *Chr*. 4A was close to the *CA1Pase* gene.

For example, we used the 2-season ASM trait (i.e., canopy-level textural changes) to perform GWAS. Under N0 treatment, 3 significant SNPs were identified on chromosome (*Chr.*) 1A, 1D and 5B using *ASM*_1−5_ (Fig. 7A, right). From 6 DAF onwards, *ASM*_6−10_, *ASM*_11−15_, and *ASM*_16−20_ were used and the 3 SNPs were also identified except their significant levels were gradually decreased: (a) the SNP on *Chr.* 1A peaked during 1 to 5 DAF; (b) the SNP on *Chr*. 1D decreased gradually during 1 to 20 DAF; and (c) the SNP on *Chr*. 5B peaked at 11 to 15 DAF (Fig. 7A, right). These findings largely correlated with key N-response phases (1 to 15 DAF) for ASM in the CGR matrix (Fig. S4). We retained significant SNPs identified across the 2 seasons (Data S8 and S9), followed by studying colocalized genes published previously (Table S14). Under N0 (i.e., without jointing N fertilizers), some known genes identified using the ASM trait included: (a) a strong signal (AX_505466150; −log10(*P*) = 5.58) was 215 kb from the *SVP* gene (*TraesCS1A01G314200*) on *Chr*. 1A, which was known for regulating inflorescence development and dormancy in wheat [78]; (b) the second strong signal (AX_289393; −log10(*P*) = 5.53) was 70 kb from the *SAR* gene (*TraesCS1D01G001700*) on *Chr*. 1D, which was reported to be involved in plant stress resistance [79]; and (c) another signal (AX_658174784; −log10(*P*) = 5.45) on *Chr*. 5B was 80 kb from *AP2L5* (i.e., *5BQ*; *TraesCS5B01G486900*), which is a flowering inhibitor that regulates the development of spikelets and florets [80].

Under N180 treatment (i.e., with jointing fertilizers), a significant SNP site associated with *ASM*_11−15_ and *ASM*_16−20_ was detected on *Chr*. 1B with its significant level peaked at 16 to 20 DAF (Fig. 7B, left). Under N270, a similar site associated with 5-d ASM phenotypic changes was observable, with a longer expression period (6 to 20 DAF) and peaked during 16 to 20 DAF (Fig. 7B, right). Notably, the genetic loci could not be identified using any static trait (i.e., *ASM*_5_, *ASM*_10_, *ASM*_15_, *ASM*_20_, and *ASM*_25_; Fig. S4E). The SNP (AX_470651294; −log10(*P*) = 5.43) identified on *Chr*. 1B was 260 kb away from the *NRT1.1A* gene (*TraesCS1B01G267900*), which is a member of the *NRT1* gene family and is known for its involvement in nitrate uptake, transport, and signal transduction [81,82].

Similarly, using other traits could also associate with some relevant genetics. For example, under N270, using *Height*_1−5_, *Height*_6−10_, *Height*_11−15_, *Height*_16−20_, and *Height*_21−25_ could identify a significant SNP on *Chr*. 3D across the 2 season during 1 to 25 DAF (Fig. 7C); and using *VARI*_11−15_ and *VARI*_16−20_ (canopy-level plant greenness and vegetation) identified significant SNPs on *Chr*. 4A during 11 to 20 DAF (2019 to 2020) and 6 to 15 DAF (2020 to 2021; Fig. 7D). The SNP identified on *Chr*. 3D (AX-597737072; −log10(*P*) = 5.57) was 273 kb away from the *CAO* gene (*TraesCS3D01G514100*), which was reported for encoding chlorophyll-a oxygenase and involving in chlorophyll-b synthesis [83]; whereas the SNP (AX-462696038; −log10(P) = 5.78) identified on *Chr*. 4A was 480 kb away from the *CA1Pase* gene (*TraesCS4A01G184100*), known for regulating CO_2_ fixation and photosynthesis to improve crop yield [84].

### Biological relevance of the study

The above GWAS analysis indicates that, in comparison with static or manually scored phenotypes at specific time points, dynamic phenotypes were likely to lead to more significant SNPs even using a small population studied in the research. This could be caused by the capture of dynamic features of N response through temporal phenotypic changes, which increased phenotypic dissimilarities between genotypes. Besides the number of SNPs, dynamic phenotypic analysis also empowered us to monitor changes of significant levels of genetic loci during N response, showing critical expression phases for the target traits, which could be valuable for in-depth genetic analysis. As N response-related gene expressions often altered during time due to external factors such as soil type, climate conditions, growth stages, and the N supply [85], it is crucial to equip researchers with the ability to use relevant traits and dynamic phenotypes to examine factors that could jointly or independently contribute to the regulation of allocating N resources at dissimilar paces.

Still, limitations encountered in the study need to be considered: (a) the key N-response phases of different traits obtained in the CGR matrix did not entirely match with the change of significant levels of the identified SNP sites; (b) according to the GWAS results, besides N0 treatment (i.e., without jointing fertilizers and hence only development related loci identified), the significant loci relevant to N utilization under N180/N270 were *CAO* (chlorophyll-a oxygenase and chlorophyll-b synthesis) and *NRT1* (nitrate uptake and transport), without other known N utilization loci. These could be caused by the small population used for GWAS or unsynchronized phenotypic and genotypic expressions (i.e., phenotypes are later than gene expression). Additional studies are needed to assess suitable traits, the calibration of phenotypic changes, and the use of a larger population with genotypically diverse lines.

### Conclusion

The ability to perform dynamic phenotypic analysis during N response is important for wheat research as it can facilitate many important topics in this field, from N uptake and N utilization, to NUE and N responsiveness. Reliable phenotypic information at specific time points (i.e., static phenotypes) or during key growth stages (dynamic phenotypes) could help researchers and breeders explore physiological and genetic mechanisms, enabling not only the identification of desirable traits but also the development of useful molecular markers to enhance N utilization and pinpoint genes of interest [86]. We present a study that measures temporal changes of 6 N response-related traits using cost-effective drones and dynamic phenotypic analysis. We incorporated morphological, spectral, and textural signals into the production of profile curves for the target traits, enabling the quantification of N-response phenotypes during 1 to 25 DAF. To study the relationship between yield performance, N utilization, and phenotypic changes, we trained an ML model called RF-NRES to classify wheat varieties into 4 N-responsiveness groups with high accuracy. After that, both static and dynamic phenotypes during N response were utilized to explore underlying genetics of N responsiveness using GWAS, demonstrating the value of dynamic phenotyping even using a small population. Coupled with plant’s N-uptake and nutrient allocation, we trust that our work could be applied in a range of N-related studies, including sustainable approaches to monitor the N status and thus N management, reliable agronomic decisions in wheat production, the large-scale screening of varieties with better N responsiveness, and optimal N applications to prevent N leaching and greenhouse gas emissions. Finally, although the main objective of this study was not to discover novel SNPs, simple adjustments to trial designs (e.g., a larger population and multiple environments) could lead to greater repetition of trials and genetic mapping, associating genetic mechanisms of N responsiveness with environment adaptation so that less agronomic inputs could be achieved to reduce environmental footprint in crop production.

## Data Availability

The source code is distributed under the Creative Commons Attribution 4.0 international license, permitting academic use, distribution, and reproduction in any medium, provided you give appropriate credit to the original authors and the source, provide a link to the Creative Commons license, and indicate if changes were made. Unless otherwise stated, the Creative Commons Public Domain Dedication (http://creativecommons.org/licenses/by/4.0) waiver applies to the data and results made available in this paper. The source code, testing data, and other datasets supporting the results presented in this article are available at https://Github.com/The-Zhou-Lab/Nitrogen-response-traits/releases. Other data and user guides are openly available upon request. The latest AirMeasurer platform can be downloaded via https://github.com/The-Zhou-Lab/UAV/releases).

## Supplementary Materials

Supplementary 1 Figs. S1 to S6 Tables S1 to S14 Notes S1 to S3
