# Supplementary material for: The Dissection of Nitrogen Response Traits Using Drone Phenotyping and Dynamic Phenotypic Analysis to Explore N Responsiveness and Associated Genetic Loci in Wheat
Source: Plant Phenomics. 2023 Dec 22;5:0128. doi: 10.34133/plantphenomics.0128 (PMC10750832; doi:10.34133/plantphenomics.0128)
Supplement: Supplementary 1 — Figs. S1 to S6 Tables S1 to S14 Notes S1 to S3 [file plantphenomics.0128.f1.docx]

**Supplementary Material**

## **Title:** The dissection of nitrogen response traits using drone phenotyping and dynamic phenotypic analysis to explore nitrogen responsiveness and associated genetic loci in wheat

## **Authors:** Guohui Ding^+^, Liyan Shen^+^, Jie Dai^+^, Robert Jackson, Shuchen Liu, Mujahid Ali, Li Sun, Mingxing Wen, Jin Xiao, Greg Deakin, Dong Jiang, Xiu-e Wang*, Ji Zhou*

+ Contributed equally

* Corresponding authors

**Table S1.** The list of 54 wheat varieties studied in the field trial.

| **Wheat varieties** | **Acronyms** | **Vernalization characteristic** | **Growing Regions** |
| --- | --- | --- | --- |
| Xumai32 | XM32 | Semi-winter | Huang-huai winter wheat region |
| Dongmai1301 | DM1301 | Semi-winter | Huang-huai winter wheat region |
| Weilai1216 | WL1216 | Semi-winter | Huang-huai winter wheat region |
| Wanmai52 | WM52 | Semi-winter | Huang-huai winter wheat region |
| Wanma108 | WM108 | Semi-winter | Huang-huai winter wheat region |
| Wanyu2 | WY2 | Semi-winter | Huang-huai winter wheat region |
| Tianmin108 | TM108 | Semi-winter | Huang-huai winter wheat region |
| Pingmai108 | PM108 | Semi-winter | Huang-huai winter wheat region |
| Lvyu098 | LY098 | Semi-winter | Huang-huai winter wheat region |
| Kenong9204 | KN9204 | Semi-winter | Huang-huai winter wheat region |
| Baoji0601 | BJ0601 | Semi-winter | Huang-huai winter wheat region |
| Aikang58 | AK58 | Semi-winter | Huang-huai winter wheat region |
| Sukemai1 | SKM1 | Springiness | Huang-huai winter wheat region |
| Huaimai30 | HM30 | Springiness | Huang-huai winter wheat region |
| Zhenmai8 | ZM8 | Springiness | Wheat region of middle and lower reaches Yangtze River |
| Zhenmai5 | ZM5 | Springiness | Wheat region of middle and lower reaches Yangtze River |
| Zhenmai4 | ZM4 | Springiness | Wheat region of middle and lower reaches Yangtze River |
| Zhenmai168 | ZM168 | Springiness | Wheat region of middle and lower reaches Yangtze River |
| Zhenmai12 | ZM12 | Springiness | Wheat region of middle and lower reaches Yangtze River |
| Zhenmai11 | ZM11 | Springiness | Wheat region of middle and lower reaches Yangtze River |
| Zhenmai10 | ZM10 | Springiness | Wheat region of middle and lower reaches Yangtze River |
| Zhenmai09196 | ZM09196 | Springiness | Wheat region of middle and lower reaches Yangtze River |
| Zhenmai9523 | ZM9523 | Springiness | Wheat region of middle and lower reaches Yangtze River |
| Yangmai25 | YM25 | Springiness | Wheat region of middle and lower reaches Yangtze River |
| Yangmai20 | YM20 | Springiness | Wheat region of middle and lower reaches Yangtze River |
| Yangmai16 | YM16 | Springiness | Wheat region of middle and lower reaches Yangtze River |
| Yangmai158 | YM158 | Springiness | Wheat region of middle and lower reaches Yangtze River |
| Yangfumai8 | YFM8 | Springiness | Wheat region of middle and lower reaches Yangtze River |
| Yangfumai6 | YFM6 | Springiness | Wheat region of middle and lower reaches Yangtze River |
| Yangfumai5 | YFM5 | Springiness | Wheat region of middle and lower reaches Yangtze River |
| Sumai553 | SM553 | Springiness | Wheat region of middle and lower reaches Yangtze River |
| P14 | P14 | Springiness | Wheat region of middle and lower reaches Yangtze River |
| P124 | P124 | Springiness | Wheat region of middle and lower reaches Yangtze River |
| CP20-39-11-2 | CP20-2 | Springiness | Wheat region of middle and lower reaches Yangtze River |
| CP20-39-11-1 | CP20-1 | Springiness | Wheat region of middle and lower reaches Yangtze River |
| CP03-28-1-1 | CP03-1 | Springiness | Wheat region of middle and lower reaches Yangtze River |
| CP02-9-4-8-2 | CP02-2 | Springiness | Wheat region of middle and lower reaches Yangtze River |
| CP02-8-5-6-1 | CP02-6-1 | Springiness | Wheat region of middle and lower reaches Yangtze River |
| CP02-8-5-5-21 | CP02-21 | Springiness | Wheat region of middle and lower reaches Yangtze River |
| CP02-63-13-1 | CP02-1 | Springiness | Wheat region of middle and lower reaches Yangtze River |
| CP02-62-1-2-2-3F10 | CP02-F10 | Springiness | Wheat region of middle and lower reaches Yangtze River |
| CP01-39-3-2-4 | CP01-4 | Springiness | Wheat region of middle and lower reaches Yangtze River |
| CP01-39-3-204 | CP01-204 | Springiness | Wheat region of middle and lower reaches Yangtze River |
| CP01-39-17-1-3 | CP01-3 | Springiness | Wheat region of middle and lower reaches Yangtze River |
| Sumai8 | SM8 | Springiness | Wheat region of middle and lower reaches Yangtze River |
| Sumai3 | SM3 | Springiness | Wheat region of middle and lower reaches Yangtze River |
| Shengxuan6 | SX6 | Springiness | Wheat region of middle and lower reaches Yangtze River |
| Ningzimai1 | NZM1 | Springiness | Wheat region of middle and lower reaches Yangtze River |
| Ningmaizi1019 | NMZ1019 | Springiness | Wheat region of middle and lower reaches Yangtze River |
| Ningmai26 | NM26 | Springiness | Wheat region of middle and lower reaches Yangtze River |
| Ningmai13 | NM13 | Springiness | Wheat region of middle and lower reaches Yangtze River |
| Nannong06Y86 | NN06Y86 | Springiness | Wheat region of middle and lower reaches Yangtze River |
| Huamai6 | HM6 | Springiness | Wheat region of middle and lower reaches Yangtze River |
| Guohong9 | GH9 | Springiness | Wheat region of middle and lower reaches Yangtze River |

**Table S2**. Soil nutrient content (0-40 cm) before sowing between 2019 and 2021 seasons.

| **Growing seasons** | **N treated block** | **Organic matter**  **（t•ha^-1^）** | **Alkali-hydrolysable N（kg•ha^-1^）** | **Inorganic N（kg•ha^-1^）** | **Available P（kg•ha^-1^）** | **Available K（kg•ha^-1^）** |
| --- | --- | --- | --- | --- | --- | --- |
|  | 1 | 158.2 | 574.76 | 65.53 | 75.24 | 345.25 |
| 2019-2020 | 2 | 129.99 | 520.86 | 69.52 | 76.23 | 352.55 |
|  | 3 | 128.58 | 503.15 | 67.72 | 68.17 | 359.05 |
|  | 1 | 109.21 | 417.03 | 51.22 | 99.14 | 402.3 |
| 2020-2021 | 2 | 118.29 | 406.27 | 51.72 | 80.82 | 427.8 |
|  | 3 | 112.43 | 393.19 | 52.6 | 85.97 | 420.5 |

**Table S3**. Evaluation of fitting functions for the six target traits to determine the best fitting method.

| **Traits** | **Evaluation metrics** | **Gaussian** | **Double Logistic** | **Fourier** | **Weibull** |
| --- | --- | --- | --- | --- | --- |
| Plant height | R^2^ | 0.98* | 0.81 | 0.92 | 0.931 |
|  | RMSE | 3.54 | 4.66 | 4.01 | 4.23 |
| Canopy coverage | R^2^ | 0.961 | 0.9 | 0.963 | 0.98* |
|  | RMSE | 0.054 | 0.064 | 0.052 | 0.032 |
| 3DCI | R2 | 0.943* | 0.667 | 0.932 | 0.497 |
|  | RMSE | 0.051 | 0.082 | 0.052 | 0.032 |
| ASM | R2 | 0.832 | \ | 0.91* | 0.44 |
|  | RMSE | 0.064 | \ | 0.039 | 0.112 |
| VARI | R2 | 0.913* | \ | 0.901 | \ |
|  | RMSE | 0.04 | \ | 0.06 | \ |
| NDYI | R2 | 0.958 | 0.69 | 0.973* | 0.55 |
|  | RMSE | 0.047 | 0.081 | 0.03 | 0.095 |
|  | | | |  |  |

Note: red-colored numbers with asterisks indicate the best correlation and hence selected fitting functions.

**Table S4**. Statistical analysis of the 12 yield-performance and N-utilization (Y&N) indices of the 54 wheat varieties under three levels of N fertilization.

| **Y&N indices** | **N treatment** | **Min value** | **Max value** | **Mean** | **Coefficient of variation (%)** |
| --- | --- | --- | --- | --- | --- |
| SNpM^2^  (Spikes per m^2^) | N0 | 234 | 384.17 | 321.68 | 12% |
|  | N180 | 302.33 | 543.17 | 416.59 | 13.6% |
|  | N270 | 353 | 588.67 | 458.29 | 15.7% |
| GNpS  (Grain number per spike) | N0 | 15.63 | 25.02 | 21.39 | 12.8% |
|  | N180 | 27.55 | 41.87 | 35.26 | 11.1% |
|  | N270 | 30.53 | 43.47 | 37.35 | 10.1% |
| TGW  Thousand Grain Weight (g) | N0 | 35.31 | 50.42 | 42.99 | 8.7% |
|  | N180 | 39.81 | 53.78 | 46.54 | 7.8% |
|  | N270 | 39.02 | 52.91 | 45.84 | 7.2% |
| GYpM^2^  Grain yield per m^2^ | N0 | 236.64 | 353.37 | 281.4 | 9.1% |
|  | N180 | 507.37 | 809.99 | 673.41 | 9% |
|  | N270 | 609.35 | 882.17 | 754.66 | 7.9% |
| Vegetative organ biomass  VOB (g•m^-2^) | N0 | 381 | 641.2 | 504.3 | 11.1% |
|  | N180 | 774.5 | 1287.5 | 1022.7 | 12.6% |
|  | N270 | 826.1 | 1442.1 | 1159.1 | 11.8% |
| Aboveground biomass  AGB (g•m^-2^) | N0 | 629.1 | 931.1 | 785.7 | 9.2% |
|  | N180 | 1338.3 | 2062.7 | 1696.1 | 9.8% |
|  | N270 | 1506.3 | 2223 | 1913.9 | 8.3% |
| Aboveground N content  AGNC (g•kg^-1^) | N0 | 5.02 | 7.19 | 6.06 | 9.2% |
|  | N180 | 8.21 | 12.98 | 10.52 | 10.8% |
|  | N270 | 9.98 | 13.52 | 11.67 | 7.3% |
| Vegetative organ N content VONC (g•kg^-1^) | N0 | 1.03 | 2.33 | 1.68 | 20.5% |
|  | N180 | 2.11 | 5.8 | 3.94 | 21.6% |
|  | N270 | 3.09 | 7.89 | 5.06 | 23.1% |
| Grain N content GNC (g•kg^-1^) | N0 | 11 | 16.45 | 13.79 | 9.6% |
|  | N180 | 14.11 | 27.33 | 20.58 | 10.9% |
|  | N270 | 17.53 | 26.8 | 21.73 | 8.3% |
| N uptake efficiency NupE (kg•kg^-1^) | N0 | 0.6 | 0.95 | 0.79 | 11.6% |
|  | N180 | 0.55 | 0.97 | 0.78 | 13.7% |
|  | N270 | 0.51 | 0.88 | 0.69 | 10.7% |
| N utilization efficiency NutE (kg•kg^-1^) | N0 | 50.07 | 66.16 | 60.2 | 9.9% |
|  | N180 | 30.69 | 46.04 | 38.08 | 11% |
|  | N270 | 25.34 | 42.25 | 34.16 | 10.8% |
| N harvest index NHI (kg•kg^-1^) | N0 | 0.75 | 0.88 | 0.82 | 4% |
|  | N180 | 0.67 | 0.85 | 0.77 | 5.9% |
|  | N270 | 0.6 | 0.84 | 0.73 | 8.2% |

**Table S5**. Eigenvalues, contribution rates, cumulative contribution rates, loading coefficients and weights of principal components of N utilization efficiency under N0 treatment

| **Y&N Indices’ principal components** | **PC1** | **PC2** | **PC3** | **PC4** | **Weights** |
| --- | --- | --- | --- | --- | --- |
| **Eigenvalue** | 4.04 | 2.84 | 2.16 | 1.49 |  |
| **Contribution rate (%)** | 33.6% | 23.7% | 18% | 12.4% |  |
| **Cumulative contribution rate (%)** | 33.6% | 57.3% | 75.3% | 87.7% |  |
| SN (spikes•m^-2^) | 0.15 | 0.26 | -0.52 | -0.03 | 7.9% |
| Grain number per spike (GNpS) | 0.11 | -0.11 | 0.48 | 0.33 | 7.2% |
| Thousand grain weight (g) | -0.04 | -0.03 | 0.37 | -0.2 | 4.2% |
| Aboveground biomass (g•m^-2^) | 0.33 | 0.17 | 0.36 | 0.26 | 9.3% |
| Vegetative organ biomass (g•m^-2^) | 0.37 | 0.3 | -0.17 | 0.19 | 9.5% |
| Grain Yield (g•m^-2^) | 0.4 | 0.29 | 0 | 0.24 | 8.9% |
| Above-ground nitrogen content (g•kg^-1^) | 0.19 | -0.52 | 0.08 | -0.08 | 8.1% |
| Vegetative organ N content (g•kg^-1^) | -0.13 | -0.42 | -0.14 | 0.48 | 8.5% |
| Grain N content (g•kg^-1^) | 0.37 | -0.28 | -0.16 | -0.32 | 9.8% |
| NupE (kg•kg^-1^) | 0.46 | -0.16 | 0.04 | 0.14 | 8.2% |
| NutE (kg•kg^-1^) | 0.31 | 0.38 | 0.26 | 0.09 | 9.5% |
| NHI (kg•kg^-1^) | 0.24 | 0.14 | 0.29 | -0.57 | 8.9% |

**Table S6**. Eigenvalues, contribution rates, cumulative contribution rates, loading coefficients and weights of principal components of N utilization efficiency under N180 treatment

| **Y&N Indices’ principal components** | PC1 | PC2 | PC3 | PC4 | Weights |
| --- | --- | --- | --- | --- | --- |
| **Eigenvalue** | 4.22 | 2.84 | 1.98 | 1.43 |  |
| **Contribution rate (%)** | 35.2% | 23.7% | 16.5% | 11.9% |  |
| **Cumulative contribution rate (%)** | 35.2% | 58.9% | 75.3% | 87.2% |  |
| SN (spikes•m^-2^) | 0.34 | -0.29 | -0.09 | -0.29 | 8.8% |
| Grain number per spike (GNpS) | -0.08 | 0.05 | 0.35 | 0.64 | 6.5% |
| Thousand grain weight (g) | -0.16 | 0.21 | 0.13 | -0.1 | 5.2% |
| Aboveground biomass (g•m^-2^) | 0.26 | -0.19 | 0.39 | 0.34 | 9% |
| Vegetative organ biomass (g•m^-2^) | 0.34 | -0.36 | 0.04 | -0.12 | 8.4% |
| Grain Yield (g•m^-2^) | 0.36 | -0.35 | 0.17 | 0.03 | 9% |
| Above-ground nitrogen content (g•kg^-1^) | 0.24 | 0.5 | 0.06 | 0.11 | 8.4% |
| Vegetative organ N content (g•kg^-1^) | 0.23 | 0.18 | -0.47 | 0.39 | 9.2% |
| Grain N content (g•kg^-1^) | 0.27 | 0.4 | 0.2 | -0.29 | 9.5% |
| NupE (kg•kg^-1^) | 0.44 | 0.14 | 0.18 | 0.09 | 8.7% |
| NutE (kg•kg^-1^) | 0.36 | 0.34 | 0.13 | 0.14 | 9.1% |
| NHI (kg•kg^-1^) | -0.16 | 0.12 | 0.6 | -0.29 | 8.1% |

**Table S7**. Eigenvalues, contribution rates, cumulative contribution rates, loading coefficients and weights of principal components of N utilization efficiency under N270 treatment

| **Y&N Indices’ principal components** | **PC1** | **PC2** | **PC3** | **PC4** | **Weights** |
| --- | --- | --- | --- | --- | --- |
| **Eigenvalue** | 4.87 | 2.50 | 2.23 | 1.23 |  |
| **Contribution rate (%)** | 40.6% | 20.8% | 18.6% | 10.3% |  |
| **Cumulative contribution rate (%)** | 40.6% | 61.4% | 80% | 90.3% |  |
| SN (spikes•m^-2^) | 0.3 | 0.4 | -0.08 | -0.15 | 8.4% |
| Grain number per spike (GNpS) | -0.18 | -0.22 | 0.18 | 0.63 | 7.6% |
| Thousand grain weight (g) | -0.24 | -0.07 | 0.37 | -0.19 | 7.2% |
| Aboveground biomass (g•m^-2^) | 0.01 | 0.37 | 0.39 | 0.45 | 7.1% |
| Vegetative organ biomass (g•m^-2^) | 0.39 | 0.24 | 0.04 | -0.07 | 7.9% |
| Grain Yield (g•m^-2^) | 0.34 | 0.35 | 0.18 | 0.11 | 9.1% |
| Above-ground nitrogen content (g•kg^-1^) | 0.21 | -0.45 | 0.29 | 0.1 | 8.6% |
| Vegetative organ N content (g•kg^-1^) | 0.31 | -0.28 | -0.26 | 0.29 | 9.4% |
| Grain N content (g•kg^-1^) | 0.17 | -0.25 | 0.45 | -0.39 | 8.7% |
| NupE (kg•kg^-1^) | 0.38 | -0.01 | 0.33 | 0.16 | 8.4% |
| NutE (kg•kg^-1^) | 0.37 | 0.32 | -0.03 | 0.17 | 8.6% |
| NHI (kg•kg^-1^) | -0.32 | 0.15 | 0.41 | -0.17 | 9% |

**Table S8**. PCA and its comprehensive scores of the 54 wheat varieties under N0 treatment

| Variety | PC1 | PC2 | PC3 | PC4 | NECS | Rank |
| --- | --- | --- | --- | --- | --- | --- |
| ZM4 | 1.85 | 1.92 | 1.28 | 0.79 | 1.4 | 1 |
| WM108 | 1.98 | 1.06 | 1.67 | -0.01 | 1.22 | 2 |
| ZM5 | 0.51 | 2.03 | 1.39 | 1.57 | 1.1 | 3 |
| ZM168 | 1.33 | 1.04 | 0.39 | 0.22 | 0.79 | 4 |
| SX6 | 0.68 | 1 | -0.52 | 0.6 | 0.45 | 5 |
| YM25 | -0.01 | -0.21 | 2.77 | 0.35 | 0.49 | 6 |
| ZM8 | 0.67 | 1.47 | 0.27 | -1.44 | 0.44 | 7 |
| DM1301 | 0.13 | 0.4 | 0.95 | 0.42 | 0.36 | 8 |
| CP02-1 | 0.25 | 0.17 | 0.3 | 0.93 | 0.29 | 9 |
| CP02-21 | 1.52 | 0.37 | -1.32 | -0.7 | 0.27 | 10 |
| WY2 | 0.56 | -0.43 | 0.94 | 0.01 | 0.26 | 11 |
| ZM9523 | 0.31 | 0.4 | 0.69 | -0.61 | 0.25 | 12 |
| CP02-2 | 0.69 | -0.74 | 0.29 | 0.62 | 0.18 | 13 |
| P124 | 1.91 | 0.57 | -2.27 | -1.54 | 0.18 | 14 |
| AK58 | 0.47 | 0.18 | -0.13 | -0.04 | 0.17 | 15 |
| KN9204 | 0.64 | -0.12 | 0 | -0.14 | 0.17 | 16 |
| WM52 | 0.14 | 0.19 | -0.17 | 0.83 | 0.16 | 17 |
| PM108 | 0.39 | -1.67 | 1.55 | 1.06 | 0.15 | 18 |
| HM6 | 0.36 | 0.77 | -0.33 | -0.78 | 0.15 | 19 |
| CP20-2 | 0.64 | -0.56 | -1.39 | 2.37 | 0.13 | 20 |
| ZM12 | 0.62 | -0.17 | 0.56 | -1.15 | 0.13 | 21 |
| CP02-F10 | -0.16 | 0.43 | -0.84 | 1.82 | 0.12 | 22 |
| YFM5 | 0.18 | 0.33 | 0.55 | -1.06 | 0.1 | 23 |
| CP03-1 | -0.02 | 0.6 | -0.57 | 0.44 | 0.09 | 24 |
| YFM8 | 1.15 | -2.56 | 0.19 | 2.19 | 0.09 | 25 |
| ZM10 | -0.13 | 0.57 | 0.28 | -0.63 | 0.06 | 26 |
| YM16 | 0.23 | 0.09 | -0.6 | 0.57 | 0.06 | 27 |
| GH9 | 0.34 | -0.84 | 0.79 | -0.19 | 0.03 | 28 |
| SKM1 | 0.07 | -0.73 | 0.59 | 0.4 | 0 | 29 |
| XM32 | 0 | 0.12 | -0.47 | -0.1 | -0.07 | 30 |
| BJ0601 | -0.07 | -0.38 | -0.93 | 1.69 | -0.07 | 31 |
| CP20-1 | 0.63 | 0.68 | -2.44 | -0.09 | -0.07 | 32 |
| P14 | 0.23 | -0.14 | 0.14 | -1.49 | -0.12 | 33 |
| CP01-3 | 0.46 | 0.47 | -2.14 | -0.32 | -0.16 | 34 |
| TM108 | -0.2 | -0.52 | -0.01 | 0.17 | -0.17 | 35 |
| YM158 | -1.53 | 1.4 | 0.47 | -0.6 | -0.17 | 36 |
| CP01-4 | -0.04 | 0.31 | -0.6 | -0.99 | -0.17 | 37 |
| NZM1 | -0.86 | -0.18 | 0.75 | -0.21 | -0.22 | 38 |
| ZM11 | -1.94 | 1.14 | 0.53 | 0.4 | -0.24 | 39 |
| CP01-204 | -0.95 | -0.21 | -0.06 | 1.1 | -0.24 | 40 |
| ZM09196 | -0.22 | -0.75 | 0.33 | -0.62 | -0.27 | 41 |
| NM13 | -0.22 | -0.42 | -0.8 | 0.04 | -0.31 | 42 |
| CP02-6-1 | -0.18 | -1.06 | 0.74 | -1.75 | -0.4 | 43 |
| NN06Y86 | -1.15 | 0.02 | -0.61 | 1.08 | -0.36 | 44 |
| HM30 | -1.34 | 0.46 | 0.96 | -1.94 | -0.41 | 45 |
| YFM6 | -1.29 | -0.82 | 0.79 | -0.2 | -0.51 | 46 |
| SM8 | -0.24 | -1.64 | 0.78 | -1.55 | -0.52 | 47 |
| YM20 | -1.02 | -0.78 | -0.05 | -0.09 | -0.55 | 48 |
| NMZ1019 | 0.45 | -1.77 | -0.69 | -1.54 | -0.58 | 49 |
| NM26 | -1.25 | -0.77 | -0.52 | 0.67 | -0.61 | 50 |
| SM3 | -3.58 | 2.63 | -0.59 | 0.54 | -0.62 | 51 |
| SM553 | -0.66 | -0.49 | -1.34 | -0.91 | -0.69 | 52 |
| WL1216 | -0.69 | -1.22 | -1.33 | -0.37 | -0.81 | 53 |
| LY098 | -1.59 | -1.64 | -0.21 | 0.16 | -0.94 | 54 |

**Table S9**. PCA and its comprehensive scores of the 54 wheat varieties under N180 treatment

| Variety | PC1 | PC2 | PC3 | PC4 | NECS | Rank |
| --- | --- | --- | --- | --- | --- | --- |
| SM8 | 3.61 | 2.79 | 0.16 | 1.44 | 2.13 | 1 |
| ZM4 | 2.42 | 3.28 | 2.09 | -0.76 | 1.88 | 2 |
| CP02-1 | 3.85 | -0.25 | 1.46 | 0.23 | 1.56 | 3 |
| ZM8 | 1.76 | -0.08 | 2.12 | 1.77 | 1.16 | 4 |
| P124 | 3.96 | -0.45 | 0.33 | -1.55 | 1.16 | 5 |
| LY098 | 1.45 | 1.98 | 0.45 | 0.58 | 1.12 | 6 |
| XM32 | 4.75 | -1.12 | -1.63 | -1.23 | 0.99 | 7 |
| ZM09196 | 0.12 | 3 | 1.68 | -0.36 | 0.99 | 8 |
| BJ0601 | 1.94 | 0.37 | -0.14 | 0.03 | 0.75 | 9 |
| CP20-2 | 0.93 | 0.62 | -0.16 | 1.17 | 0.59 | 10 |
| SX6 | 2.41 | -1.46 | -0.56 | 1.38 | 0.57 | 11 |
| TM108 | 1.92 | -0.21 | -1.99 | 2.03 | 0.54 | 12 |
| PM108 | -2.05 | 3.19 | 1.89 | 1.48 | 0.52 | 13 |
| YM20 | -0.13 | 0.33 | 2.34 | 0.61 | 0.49 | 14 |
| NN06Y86 | 1.33 | -0.41 | -0.18 | 1.25 | 0.49 | 15 |
| ZM168 | 0.41 | 0.71 | 1.42 | -0.94 | 0.44 | 16 |
| SM3 | 1.92 | -0.71 | -0.72 | 0.29 | 0.42 | 17 |
| KN9204 | 1.37 | 1.36 | -1.66 | -1.08 | 0.4 | 18 |
| ZM9523 | 0.74 | -0.37 | 0.87 | -0.46 | 0.26 | 19 |
| ZM12 | -0.23 | 1.41 | 1.29 | -1.32 | 0.31 | 20 |
| WY2 | 0.94 | 0.99 | -0.84 | -1.42 | 0.26 | 21 |
| P14 | 0.51 | 0.72 | 0.09 | -1.37 | 0.2 | 22 |
| WM108 | 0.73 | -1.71 | 2.32 | -0.53 | 0.17 | 23 |
| HM6 | 1.06 | -0.97 | 0.13 | 0 | 0.16 | 24 |
| YFM6 | 0.16 | 0.04 | 1.46 | -1.3 | 0.15 | 25 |
| ZM5 | 0.03 | -1.42 | 2 | 0.43 | 0.05 | 26 |
| CP02-6-1 | -1.54 | 1.22 | 0.92 | 0.27 | -0.07 | 27 |
| DM1301 | -0.03 | 0.54 | -1.5 | 1.1 | 0 | 28 |
| NZM1 | -1.3 | 3.79 | -3.45 | 0.17 | -0.11 | 29 |
| CP20-1 | 0.5 | -0.74 | 0.08 | -1.33 | -0.14 | 30 |
| SKM1 | -0.76 | 0.74 | -0.85 | 0.45 | -0.18 | 31 |
| CP01-204 | 0.02 | -0.2 | -0.77 | -0.26 | -0.2 | 32 |
| CP01-4 | 0.81 | -0.06 | -1.57 | -1.78 | -0.2 | 33 |
| SM553 | -1.56 | 1.89 | 0 | -1 | -0.22 | 34 |
| CP01-3 | 1.28 | -2.29 | -1.65 | 0.38 | -0.32 | 35 |
| NM26 | -0.22 | -0.38 | -1.63 | 1.8 | -0.22 | 36 |
| ZM10 | -0.79 | -0.25 | 0.69 | -0.8 | -0.32 | 37 |
| ZM11 | -0.24 | -1.77 | 0.77 | 0.33 | -0.34 | 38 |
| NM13 | -1.22 | 0.97 | -2.24 | 1.6 | -0.38 | 39 |
| GH9 | -0.62 | -2.07 | 1.06 | 1.31 | -0.38 | 40 |
| WL1216 | -2.23 | 1.93 | -0.19 | -0.27 | -0.39 | 41 |
| YM25 | -1.73 | -1.3 | 1.93 | 1.22 | -0.45 | 42 |
| YFM8 | -1.43 | -1.12 | 1.09 | 1.07 | -0.46 | 43 |
| NMZ1019 | -1.32 | -0.2 | -0.9 | 0.84 | -0.56 | 44 |
| CP02-F10 | 0.56 | -2.23 | -1.67 | 0.13 | -0.59 | 45 |
| CP03-1 | -1.02 | 0.53 | -2.57 | -0.8 | -0.75 | 46 |
| YM16 | -2.72 | -1.24 | 0.99 | 1.46 | -0.92 | 47 |
| CP02-21 | 1.17 | -4.12 | -0.15 | -3.33 | -0.98 | 48 |
| YFM5 | -3.58 | 1.63 | -0.9 | 0.12 | -1.01 | 49 |
| AK58 | -2.66 | 0.11 | -1.84 | -1.35 | -1.37 | 50 |
| CP02-2 | -1.97 | -3.93 | -1.05 | 1.88 | -1.57 | 51 |
| HM30 | -4.64 | 0.83 | 0.11 | -1.89 | -1.64 | 52 |
| YM158 | -4.91 | -1.43 | 1.32 | -0.98 | -1.97 | 53 |
| WM52 | -3.73 | -2.45 | -0.29 | -0.71 | -2.02 | 54 |

**Table S10.** PCA and its comprehensive scores of the 54 wheat varieties under N270 treatment

| Variety | PC1 | PC2 | PC3 | PC4 | NECS | Rank |
| --- | --- | --- | --- | --- | --- | --- |
| CP02-21 | 7.51 | 3.67 | -0.74 | -1.63 | 3.51 | 1 |
| BJ0601 | 3.46 | -0.93 | 2.84 | 0.19 | 1.76 | 2 |
| XM32 | 4.29 | -0.02 | -0.32 | 0.42 | 1.72 | 3 |
| CP02-1 | 2.42 | 1.7 | 0.81 | 1.11 | 1.6 | 4 |
| CP03-1 | 4.42 | -2.27 | -1.34 | 0.01 | 1.07 | 5 |
| KN9204 | 2.58 | -0.39 | 0.64 | -0.89 | 0.99 | 6 |
| ZM9523 | 1.97 | -0.65 | 1.14 | 0.76 | 0.95 | 7 |
| P124 | 1.7 | 1.46 | 0.39 | -1.23 | 0.94 | 8 |
| ZM5 | 1.23 | 0.56 | 1.27 | -0.21 | 0.83 | 9 |
| ZM8 | 0.61 | -0.06 | 2.45 | 1.35 | 0.83 | 10 |
| CP20-1 | 3.02 | 0.94 | -3.27 | 0.03 | 0.82 | 11 |
| CP02-F10 | 0.72 | 2.39 | -0.29 | 0.7 | 0.81 | 12 |
| CP20-2 | 0.7 | 0.46 | 0.26 | 1.06 | 0.54 | 13 |
| NZM1 | 1.42 | -0.58 | 0.72 | -0.74 | 0.51 | 14 |
| YM25 | -0.91 | 1.78 | 1.92 | 1.42 | 0.5 | 15 |
| P14 | 1.03 | -0.47 | 0.99 | -0.32 | 0.47 | 16 |
| CP01-4 | -0.79 | 3.56 | 0.16 | 0.12 | 0.46 | 17 |
| WM108 | -0.27 | 2.23 | 0.79 | -0.48 | 0.45 | 18 |
| SM3 | 1.71 | -1.94 | 0.77 | -0.01 | 0.43 | 19 |
| HM6 | 0.26 | 0.62 | 0.62 | 0.16 | 0.37 | 20 |
| ZM10 | 0.89 | -2.15 | 1.2 | 1.84 | 0.32 | 21 |
| ZM168 | 0.32 | -0.25 | 0.24 | 1.46 | 0.27 | 22 |
| CP02-6-1 | 0.49 | -2.02 | 3.47 | -1.76 | 0.24 | 23 |
| CP01-204 | -0.28 | 0.74 | 0.35 | -0.09 | 0.1 | 24 |
| GH9 | 0.68 | -0.47 | -0.59 | -0.07 | 0.06 | 25 |
| AK58 | 0.05 | 1.61 | -0.92 | -1.8 | 0 | 26 |
| ZM12 | -1.7 | 0.4 | 3.4 | -0.33 | -0.01 | 27 |
| NN06Y86 | 0.94 | -1.5 | -1.89 | 1.56 | -0.12 | 28 |
| ZM11 | 1.2 | -1.97 | -1.6 | 0.45 | -0.17 | 29 |
| YM158 | -2.31 | 1.22 | 2.15 | 0.96 | -0.19 | 30 |
| SX6 | -0.91 | 2.29 | -2.09 | 0.77 | -0.2 | 31 |
| NM13 | -0.71 | 0.48 | -0.73 | 1.13 | -0.21 | 32 |
| WM52 | 0.81 | -1.88 | -0.84 | -0.6 | -0.28 | 33 |
| CP02-2 | -1.04 | -0.12 | -0.48 | 2.15 | -0.31 | 34 |
| SM553 | 0.19 | -0.67 | -0.29 | -2.17 | -0.34 | 35 |
| NM26 | -0.79 | 1.74 | -3.09 | 0.99 | -0.43 | 36 |
| TM108 | -2.27 | 2.14 | -0.52 | -0.05 | -0.57 | 37 |
| YM16 | -1.66 | 0.84 | 0.27 | -0.37 | -0.49 | 38 |
| YM20 | -1.31 | -1.34 | 0.15 | 1.52 | -0.63 | 39 |
| YFM6 | -0.76 | -0.47 | -1.15 | -0.45 | -0.67 | 40 |
| SKM1 | 0.9 | -3.07 | -2.71 | 0.7 | -0.71 | 41 |
| NMZ1019 | -0.56 | -1.85 | -0.71 | -0.1 | -0.75 | 42 |
| SM8 | -2.44 | 0.52 | 0.22 | 0.83 | -0.75 | 43 |
| ZM4 | -2.23 | -0.88 | 1.58 | 0.14 | -0.78 | 44 |
| YFM8 | -1.9 | -1.67 | 0.79 | 0.92 | -0.88 | 45 |
| WL1216 | -1.71 | -0.7 | 1.03 | -2.31 | -0.89 | 46 |
| CP01-3 | 0.12 | -2.14 | -2.12 | -1.39 | -0.93 | 47 |
| YFM5 | -2.71 | 0.17 | 0.76 | -0.39 | -0.96 | 48 |
| WY2 | -2.45 | 1.8 | -0.72 | -2.56 | -1.02 | 49 |
| DM1301 | -3.47 | 1.83 | -1.18 | 0.9 | -1.15 | 50 |
| PM108 | -2.64 | -0.53 | -0.78 | -0.89 | -1.42 | 51 |
| LY098 | -2.22 | -1.49 | -0.97 | -0.65 | -1.46 | 52 |
| HM30 | -2.99 | -1.92 | 0.21 | -1.7 | -1.75 | 53 |
| ZM09196 | -4.66 | -0.71 | -2.21 | -0.46 | -2.5 | 54 |

**Table S11.** A matrix of weights for nitrogen response related traits.

| **Day after fertilization (DAF)** | **Trait-based weights** | | | | | |
| --- | --- | --- | --- | --- | --- | --- |
|  | Height | Coverage | 3DCI | ASM | VARI | NDYI |
| 1-5DAF | 5.08% | 5.27% | 3.91% | 4.89% | 4.62% | 4.42% |
| 6-10DAF | 6.72% | 6.31% | 5.73% | 7.11% | 6.10% | 6.18% |
| 11-15DAF | 4.60% | 4.25% | 3.70% | 4.55% | 4.14% | 4.39% |
| 16-20DAF | 2.59% | 2.66% | 2.59% | 2.84% | 2.42% | 2.97% |
| 21-25DAF | 1.60% | 1.65% | 1.68% | 1.66% | 1.47% | 1.95% |
| **Total trait weights** | 20.58% | 20.15% | 17.61% | 21.05% | 18.76% | 19.91% |
| **Compound** **weights** | 17.43% | 17.06% | 17.83% | 14.92% | 15.89% | 16.86% |

**Table S12**. Number, physical distance coverage and marker density of SNPs on the 21 wheat Chromosomes and in the three sub-genomes

| **Chromosome** | **Number of SNPs** | **Coverage physical**  **region (Mb)** | **Density (SNP/Mb)** |
| --- | --- | --- | --- |
| 1A | 2468 | 592.1 | 4.168 |
| 1B | 2409 | 687.4 | 3.505 |
| 1D | 1903 | 495.2 | 3.843 |
| 2A | 2574 | 780.5 | 3.298 |
| 2B | 2405 | 800.9 | 3.003 |
| 2D | 1738 | 649.2 | 2.677 |
| 3A | 1998 | 750.5 | 2.662 |
| 3B | 2519 | 829.5 | 3.037 |
| 3D | 1478 | 615 | 2.403 |
| 4A | 1994 | 739.5 | 2.696 |
| 4B | 2474 | 672.9 | 3.677 |
| 4D | 727 | 508.8 | 1.429 |
| 5A | 2507 | 708.9 | 3.536 |
| 5B | 2525 | 712.4 | 3.544 |
| 5D | 1480 | 564.4 | 2.622 |
| 6A | 2449 | 617.5 | 3.966 |
| 6B | 2387 | 719.3 | 3.319 |
| 6D | 1459 | 473.5 | 3.081 |
| 7A | 2438 | 734.3 | 3.32 |
| 7B | 2269 | 750.5 | 3.023 |
| 7D | 2014 | 638.4 | 3.155 |
| **Genome A** | 16428 | 4923.3 | 3.378 |
| **Genome B** | 16988 | 5172.9 | 3.301 |
| **Genome D** | 10799 | 3944.5 | 2.744 |
| **Total** | 44215 | 14040.7 | 3.141 (*avg.*) |

**Table S13**. Significant SNPs identified using the N-response traits across two seasons.

| **Year** | **N treatments** | **Traits CGRs** | **DAF** | **Chromosome** | **Position (bp)** | ***P*-value (GLM)** |
| --- | --- | --- | --- | --- | --- | --- |
| 2021 | N0 | ASM | 6-10DAF | Chr. 1D | 289393 | 5.525 |
| 2021 | N0 | ASM | 1-5DAF | Chr. 1D | 289393 | 5.469 |
| 2021 | N0 | ASM | 11-15DAF | Chr. 1D | 289393 | 5.361 |
| 2020 | N0 | ASM | 6-10DAF | Chr. 1D | 289393 | 5.147 |
| 2020 | N0 | ASM | 11-15DAF | Chr. 1D | 289393 | 5.128 |
| 2021 | N0 | ASM | 1-5DAF | Chr. 1D | 296305 | 5.469 |
| 2020 | N0 | ASM | 6-10DAF | Chr. 1D | 296305 | 5.147 |
| 2021 | N0 | ASM | 6-10DAF | Chr. 1D | 296305 | 5.525 |
| 2020 | N0 | ASM | 11-15DAF | Chr. 1D | 296305 | 5.128 |
| 2021 | N0 | ASM | 11-15DAF | Chr. 1D | 296305 | 5.361 |
| 2021 | N0 | ASM | 1-5DAF | Chr. 1D | 304095 | 5.469 |
| 2020 | N0 | ASM | 6-10DAF | Chr. 1D | 304095 | 5.147 |
| 2021 | N0 | ASM | 6-10DAF | Chr. 1D | 304095 | 5.525 |
| 2020 | N0 | ASM | 11-15DAF | Chr. 1D | 304095 | 5.128 |
| 2021 | N0 | ASM | 11-15DAF | Chr. 1D | 304095 | 5.361 |
| 2021 | N0 | ASM | 1-5DAF | Chr. 1D | 322383 | 5.469 |
| 2020 | N0 | ASM | 6-10DAF | Chr. 1D | 322383 | 5.147 |
| 2021 | N0 | ASM | 6-10DAF | Chr. 1D | 322383 | 5.525 |
| 2020 | N0 | ASM | 11-15DAF | Chr. 1D | 322383 | 5.128 |
| 2021 | N0 | ASM | 11-15DAF | Chr. 1D | 322383 | 5.361 |
| 2021 | N0 | ASM | 1-5DAF | Chr. 1D | 329117 | 5.036 |
| 2020 | N0 | ASM | 6-10DAF | Chr. 1D | 329117 | 5.134 |
| 2021 | N0 | ASM | 6-10DAF | Chr. 1D | 329117 | 5.203 |
| 2020 | N0 | ASM | 11-15DAF | Chr. 1D | 329117 | 5.203 |
| 2021 | N0 | ASM | 11-15DAF | Chr. 1D | 329117 | 5.196 |
| 2021 | N0 | ASM | 1-5DAF | Chr. 1D | 330256 | 5.469 |
| 2020 | N0 | ASM | 6-10DAF | Chr. 1D | 330256 | 5.147 |
| 2021 | N0 | ASM | 6-10DAF | Chr. 1D | 330256 | 5.525 |
| 2020 | N0 | ASM | 11-15DAF | Chr. 1D | 330256 | 5.128 |
| 2021 | N0 | ASM | 11-15DAF | Chr. 1D | 330256 | 5.361 |
| 2021 | N0 | ASM | 1-5DAF | Chr. 1D | 333633 | 5.469 |
| 2020 | N0 | ASM | 6-10DAF | Chr. 1D | 333633 | 5.147 |
| 2021 | N0 | ASM | 6-10DAF | Chr. 1D | 333633 | 5.525 |
| 2020 | N0 | ASM | 11-15DAF | Chr. 1D | 333633 | 5.128 |
| 2021 | N0 | ASM | 11-15DAF | Chr. 1D | 333633 | 5.361 |
| 2021 | N0 | ASM | 1-5DAF | Chr. 1D | 342524 | 5.469 |
| 2020 | N0 | ASM | 6-10DAF | Chr. 1D | 342524 | 5.147 |
| 2021 | N0 | ASM | 6-10DAF | Chr. 1D | 342524 | 5.525 |
| 2020 | N0 | ASM | 11-15DAF | Chr. 1D | 342524 | 5.128 |
| 2021 | N0 | ASM | 11-15DAF | Chr. 1D | 342524 | 5.361 |
| 2021 | N0 | ASM | 1-5DAF | Chr. 1D | 384215 | 5.01 |
| 2021 | N0 | ASM | 6-10DAF | Chr. 1D | 384215 | 5.187 |
| 2020 | N0 | ASM | 11-15DAF | Chr. 1D | 384215 | 5.044 |
| 2021 | N0 | ASM | 11-15DAF | Chr. 1D | 384215 | 5.186 |
| 2021 | N0 | ASM | 1-5DAF | Chr. 1D | 388375 | 5.469 |
| 2020 | N0 | ASM | 6-10DAF | Chr. 1D | 388375 | 5.147 |
| 2021 | N0 | ASM | 6-10DAF | Chr. 1D | 388375 | 5.525 |
| 2020 | N0 | ASM | 11-15DAF | Chr. 1D | 388375 | 5.128 |
| 2021 | N0 | ASM | 11-15DAF | Chr. 1D | 388375 | 5.361 |
| 2020 | N0 | ASM | 1-5DAF | Chr. 1D | 413871 | 5.469 |
| 2021 | N0 | ASM | 1-5DAF | Chr. 1D | 413871 | 5.447 |
| 2020 | N0 | ASM | 6-10DAF | Chr. 1D | 413871 | 5.414 |
| 2021 | N0 | ASM | 6-10DAF | Chr. 1D | 413871 | 5.296 |
| 2020 | N0 | ASM | 11-15DAF | Chr. 1D | 413871 | 5.198 |
| 2020 | N0 | ASM | 1-5DAF | Chr. 1D | 20067129 | 5.388 |
| 2021 | N0 | ASM | 1-5DAF | Chr. 1D | 20067129 | 5.682 |
| 2020 | N0 | ASM | 6-10DAF | Chr. 1D | 20067129 | 5.198 |
| 2021 | N0 | ASM | 6-10DAF | Chr. 1D | 20067129 | 5.39 |
| 2020 | N0 | ASM | 1-5DAF | Chr. 1D | 20087203 | 5.171 |
| 2021 | N0 | ASM | 1-5DAF | Chr. 1D | 20087203 | 5.328 |
| 2020 | N0 | ASM | 6-10DAF | Chr. 1D | 20087203 | 5.102 |
| 2021 | N0 | ASM | 6-10DAF | Chr. 1D | 20087203 | 5.18 |
| 2020 | N0 | ASM | 1-5DAF | Chr. 1D | 20112870 | 5.171 |
| 2021 | N0 | ASM | 1-5DAF | Chr. 1D | 20112870 | 5.328 |
| 2020 | N0 | ASM | 6-10DAF | Chr. 1D | 20112870 | 5.102 |
| 2021 | N0 | ASM | 6-10DAF | Chr. 1D | 20112870 | 5.18 |
| 2020 | N0 | ASM | 1-5DAF | Chr. 1D | 20202872 | 5.388 |
| 2021 | N0 | ASM | 1-5DAF | Chr. 1D | 20202872 | 5.682 |
| 2020 | N0 | ASM | 6-10DAF | Chr. 1D | 20202872 | 5.198 |
| 2021 | N0 | ASM | 6-10DAF | Chr. 1D | 20202872 | 5.39 |
| 2020 | N0 | ASM | 1-5DAF | Chr. 1D | 20302095 | 5.171 |
| 2021 | N0 | ASM | 1-5DAF | Chr. 1D | 20302095 | 5.328 |
| 2020 | N0 | ASM | 6-10DAF | Chr. 1D | 20302095 | 5.102 |
| 2021 | N0 | ASM | 6-10DAF | Chr. 1D | 20302095 | 5.18 |
| 2020 | N0 | ASM | 1-5DAF | Chr. 1D | 20306532 | 5.388 |
| 2021 | N0 | ASM | 1-5DAF | Chr. 1D | 20306532 | 5.682 |
| 2020 | N0 | ASM | 6-10DAF | Chr. 1D | 20306532 | 5.198 |
| 2021 | N0 | ASM | 6-10DAF | Chr. 1D | 20306532 | 5.39 |
| 2020 | N0 | ASM | 1-5DAF | Chr. 1D | 20307956 | 5.388 |
| 2021 | N0 | ASM | 1-5DAF | Chr. 1D | 20307956 | 5.682 |
| 2020 | N0 | ASM | 6-10DAF | Chr. 1D | 20307956 | 5.198 |
| 2021 | N0 | ASM | 6-10DAF | Chr. 1D | 20307956 | 5.39 |
| 2020 | N180 | ASM | 16-20DAF | Chr. 1B | 470538069 | 5.196 |
| 2021 | N180 | ASM | 16-20DAF | Chr. 1B | 470538069 | 5.196 |
| 2020 | N270 | ASM | 16-20DAF | Chr. 1B | 470538069 | 5.33 |
| 2021 | N270 | ASM | 16-20DAF | Chr. 1B | 470538069 | 5.393 |
| 2020 | N180 | ASM | 11-15DAF | Chr. 1B | 470599759 | 5.161 |
| 2021 | N180 | ASM | 11-15DAF | Chr. 1B | 470599759 | 5.127 |
| 2020 | N180 | ASM | 16-20DAF | Chr. 1B | 470599759 | 5.435 |
| 2021 | N180 | ASM | 16-20DAF | Chr. 1B | 470599759 | 5.218 |
| 2020 | N270 | ASM | 11-15DAF | Chr. 1B | 470599759 | 5.221 |
| 2020 | N270 | ASM | 16-20DAF | Chr. 1B | 470599759 | 5.221 |
| 2020 | N270 | ASM | 6-10DAF | Chr. 1B | 470599759 | 5.176 |
| 2021 | N270 | ASM | 11-15DAF | Chr. 1B | 470599759 | 5.22 |
| 2021 | N270 | ASM | 16-20DAF | Chr. 1B | 470599759 | 5.221 |
| 2021 | N270 | ASM | 6-10DAF | Chr. 1B | 470599759 | 5.176 |
| 2020 | N180 | ASM | 11-15DAF | Chr. 1B | 470651294 | 5.161 |
| 2021 | N180 | ASM | 11-15DAF | Chr. 1B | 470651294 | 5.127 |
| 2020 | N180 | ASM | 16-20DAF | Chr. 1B | 470651294 | 5.435 |
| 2021 | N180 | ASM | 16-20DAF | Chr. 1B | 470651294 | 5.218 |
| 2020 | N270 | ASM | 11-15DAF | Chr. 1B | 470651294 | 5.217 |
| 2020 | N270 | ASM | 16-20DAF | Chr. 1B | 470651294 | 5.217 |
| 2020 | N270 | ASM | 6-10DAF | Chr. 1B | 470651294 | 5.161 |
| 2021 | N270 | ASM | 11-15DAF | Chr. 1B | 470651294 | 5.221 |
| 2021 | N270 | ASM | 16-20DAF | Chr. 1B | 470651294 | 5.221 |
| 2021 | N270 | ASM | 6-10DAF | Chr. 1B | 470651294 | 5.161 |
| 2020 | N0 | ASM | 1-5DAF | Chr. 1A | 504631165 | 5.132 |
| 2021 | N0 | ASM | 1-5DAF | Chr. 1A | 504631165 | 5.186 |
| 2020 | N0 | ASM | 1-5DAF | Chr. 1A | 504704344 | 5.132 |
| 2021 | N0 | ASM | 1-5DAF | Chr. 1A | 504704344 | 5.186 |
| 2020 | N0 | ASM | 1-5DAF | Chr. 1A | 504825009 | 5.132 |
| 2021 | N0 | ASM | 1-5DAF | Chr. 1A | 504825009 | 5.186 |
| 2020 | N0 | ASM | 1-5DAF | Chr. 1A | 504923792 | 5.132 |
| 2021 | N0 | ASM | 1-5DAF | Chr. 1A | 504923792 | 5.186 |
| 2020 | N0 | ASM | 1-5DAF | Chr. 1A | 504983532 | 5.132 |
| 2021 | N0 | ASM | 1-5DAF | Chr. 1A | 504983532 | 5.186 |
| 2020 | N0 | ASM | 1-5DAF | Chr. 1A | 504991473 | 5.132 |
| 2021 | N0 | ASM | 1-5DAF | Chr. 1A | 504991473 | 5.186 |
| 2020 | N0 | ASM | 1-5DAF | Chr. 1A | 505061276 | 5.132 |
| 2021 | N0 | ASM | 1-5DAF | Chr. 1A | 505061276 | 5.186 |
| 2020 | N0 | ASM | 1-5DAF | Chr. 1A | 505070401 | 5.581 |
| 2021 | N0 | ASM | 1-5DAF | Chr. 1A | 505070401 | 5.467 |
| 2020 | N0 | ASM | 1-5DAF | Chr. 1A | 505093212 | 5.132 |
| 2021 | N0 | ASM | 1-5DAF | Chr. 1A | 505093212 | 5.186 |
| 2020 | N0 | ASM | 1-5DAF | Chr. 1A | 505129063 | 5.581 |
| 2021 | N0 | ASM | 1-5DAF | Chr. 1A | 505129063 | 5.467 |
| 2020 | N0 | ASM | 1-5DAF | Chr. 1A | 505337840 | 5.132 |
| 2021 | N0 | ASM | 1-5DAF | Chr. 1A | 505337840 | 5.186 |
| 2020 | N0 | ASM | 1-5DAF | Chr. 1A | 505466150 | 5.581 |
| 2021 | N0 | ASM | 1-5DAF | Chr. 1A | 505466150 | 5.467 |
| 2020 | N0 | ASM | 11-15DAF | Chr. 5B | 658174784 | 5.452 |
| 2020 | N0 | ASM | 6-10DAF | Chr. 5B | 658174784 | 5.341 |
| 2020 | N0 | ASM | 16-20DAF | Chr. 5B | 658174784 | 5.129 |
| 2020 | N0 | ASM | 1-5DAF | Chr. 5B | 658174784 | 5.031 |
| 2021 | N0 | ASM | 11-15DAF | Chr. 5B | 658174784 | 5.452 |
| 2021 | N0 | ASM | 6-10DAF | Chr. 5B | 658174784 | 5.341 |
| 2021 | N0 | ASM | 16-20DAF | Chr. 5B | 658174784 | 5.129 |
| 2021 | N0 | ASM | 1-5DAF | Chr. 5B | 658174784 | 5.031 |
| 2020 | N270 | Height | 1-5DAF | Chr. 3D | 597737072 | 5.104 |
| 2021 | N270 | Height | 1-5DAF | Chr. 3D | 597737072 | 5.24 |
| 2020 | N270 | Height | 6-10DAF | Chr. 3D | 597737072 | 5.663 |
| 2021 | N270 | Height | 6-10DAF | Chr. 3D | 597737072 | 5.715 |
| 2020 | N270 | Height | 11-15DAF | Chr. 3D | 597737072 | 5.81 |
| 2021 | N270 | Height | 11-15DAF | Chr. 3D | 597737072 | 5.766 |
| 2020 | N270 | Height | 16-20DAF | Chr. 3D | 597737072 | 5.649 |
| 2021 | N270 | Height | 16-20DAF | Chr. 3D | 597737072 | 5.536 |
| 2020 | N270 | Height | 21-25DAF | Chr. 3D | 597737072 | 5.297 |
| 2021 | N270 | Height | 21-25DAF | Chr. 3D | 597737072 | 5.151 |
| 2020 | N270 | Height | 1-5DAF | Chr. 3D | 603677636 | 5.104 |
| 2021 | N270 | Height | 1-5DAF | Chr. 3D | 603677636 | 5.24 |
| 2020 | N270 | Height | 6-10DAF | Chr. 3D | 603677636 | 5.663 |
| 2021 | N270 | Height | 6-10DAF | Chr. 3D | 603677636 | 5.715 |
| 2020 | N270 | Height | 11-15DAF | Chr. 3D | 603677636 | 5.81 |
| 2021 | N270 | Height | 11-15DAF | Chr. 3D | 603677636 | 5.766 |
| 2020 | N270 | Height | 16-20DAF | Chr. 3D | 603677636 | 5.649 |
| 2021 | N270 | Height | 16-20DAF | Chr. 3D | 603677636 | 5.536 |
| 2020 | N270 | Height | 21-25DAF | Chr. 3D | 603677636 | 5.297 |
| 2021 | N270 | Height | 21-25DAF | Chr. 3D | 603677636 | 5.151 |
| 2020 | N270 | VARI | 11-15DAF | Chr. 4A | 462696038 | 5.695 |
| 2021 | N270 | VARI | 11-15DAF | Chr. 4A | 462696038 | 5.785 |
| 2020 | N270 | VARI | 11-15DAF | Chr. 4A | 468750775 | 5.884 |
| 2021 | N270 | VARI | 11-15DAF | Chr. 4A | 468750775 | 5.948 |
| 2020 | N270 | VARI | 11-15DAF | Chr. 4A | 472932565 | 5.695 |
| 2021 | N270 | VARI | 11-15DAF | Chr. 4A | 472932565 | 5.785 |
| 2020 | N270 | VARI | 11-15DAF | Chr. 4A | 476976750 | 5.695 |
| 2021 | N270 | VARI | 11-15DAF | Chr. 4A | 476976750 | 5.785 |
| 2020 | N270 | VARI | 11-15DAF | Chr. 4A | 479806413 | 5.695 |
| 2021 | N270 | VARI | 11-15DAF | Chr. 4A | 479806413 | 5.785 |
| 2020 | N270 | VARI | 11-15DAF | Chr. 4A | 497655031 | 5.695 |
| 2021 | N270 | VARI | 11-15DAF | Chr. 4A | 497655031 | 5.785 |
| 2020 | N270 | VARI | 16-20DAF | Chr. 4A | 468750775 | 5.016 |
| 2020 | N270 | VARI | 6-10DAF | Chr. 7D | 591388815 | 5.036 |
| 2020 | N180 | VARI | 6-10DAF | Chr. 7D | 591388815 | 5.176 |
| 2021 | N180 | VARI | 6-10DAF | Chr. 7D | 591388815 | 5.007 |
| 2021 | N270 | VARI | 6-10DAF | Chr*.* 7D | 591388815 | 5.135 |

**Table S14.** GWAS-identified significant loci and their co-localized genes with reported functions.

| **Trait** | **Chr.** | **SNP** | **Distance**  **(kb)** | **-*log_10_P*** | **Candidate gene ID** | **Description** |
| --- | --- | --- | --- | --- | --- | --- |
| ASM | 1A | AX-505466150 | 214.644 | 5.581 | TraesCS1A01G314200 | Ethylene-responsive transcription factor (SVP) |
| ASM | 1B | AX-470599759 | 311.141 | 5.434 | TraesCS1B01G267900 | Protein NRT1/ PTR FAMILY 1.1 |
| ASM | 1B | AX-470599759 | 424.665 | 5.434 | TraesCS1B01G268200 | Protein NRT1/ PTR FAMILY 1.1 |
| ASM | 1B | AX-470651294 | 259.606 | 5.434 | TraesCS1B01G267900 | Protein NRT1/ PTR FAMILY 1.1 |
| ASM | 1B | AX-470651294 | 373.13 | 5.434 | TraesCS1B01G268200 | Protein NRT1/ PTR FAMILY 1.1 |
| ASM | 1D | AX-289393 | 70.066 | 5.525 | TraesCS1D01G001700 | Receptor protein kinase (SAR) |
| Height | 3D | AX-597737072 | 272.921 | 5.766 | TraesCS3D01G514100 | Chlorophyllide a oxygenase (CAO genes) |
| VARI | 4A | AX-462696038 | 479.97 | 5.784 | TraesCS4A01G184100 | 3-bisphosphoglycerate-dependent phosphoglyce-rate mutase (CA1Pase gene) |
| ASM | 5B | AX-658174784 | 79.718 | 5.451 | TraesCS5B01G486900 | Ethylene-responsive transcription factor (AP2L5) 5Bq gene |

**Figure S1.** Correlation analysis to verify AirMeasurer-derived canopy height and canopy coverage traits using 486 wheat plots in the 2019-2020 season.


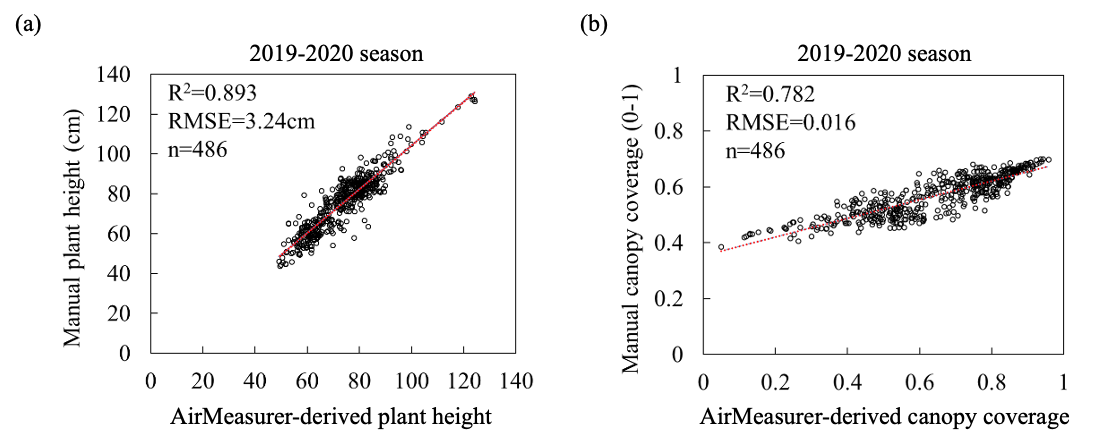


(a) Correlation analysis conducted between manually measured canopy plant height and AirMeasurer-derived plant height measures. (b) Correlation analysis conducted between manually scored canopy coverage and AirMeasurer-derived canopy coverage.

**Figure S2.** Differences of the 12 yield-performance and N-utilization indices measured under the three levels of N fertilization between 2019 and 2021 seasons.


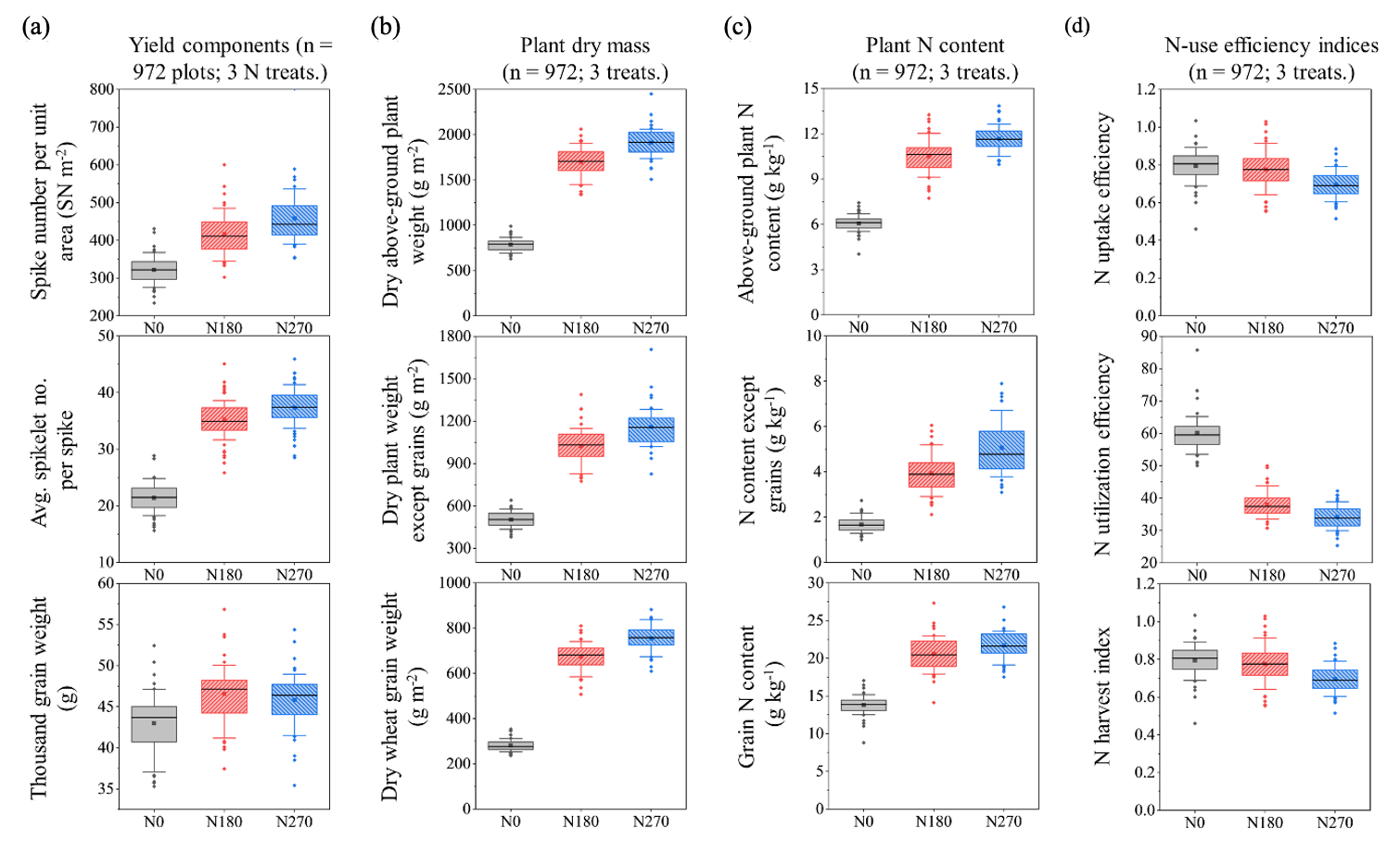


(a) The key yield components including spike number per unit area (SNpM^2^), grain number per spike (GNpS), and thousand grain weight. (b) Plant dry biomass including aboveground biomass (g·m^-2^), aboveground straw biomass (g·m^-2^), grain yield (g·m^-2^). (c) Plant N content including N in aboveground biomass (g·kg^-1^), N in straw (g·kg^-1^), grain N content (g·kg^-1^). (d) The N efficiency indices: the nitrogen uptake efficiency (NUpE), the nitrogen utilization efficiency (NUtE), the nitrogen harvest index (NHI).

**Figure S3.** Phenotypic changes of the six target traits under three N treatments in the 2020-2021 season.


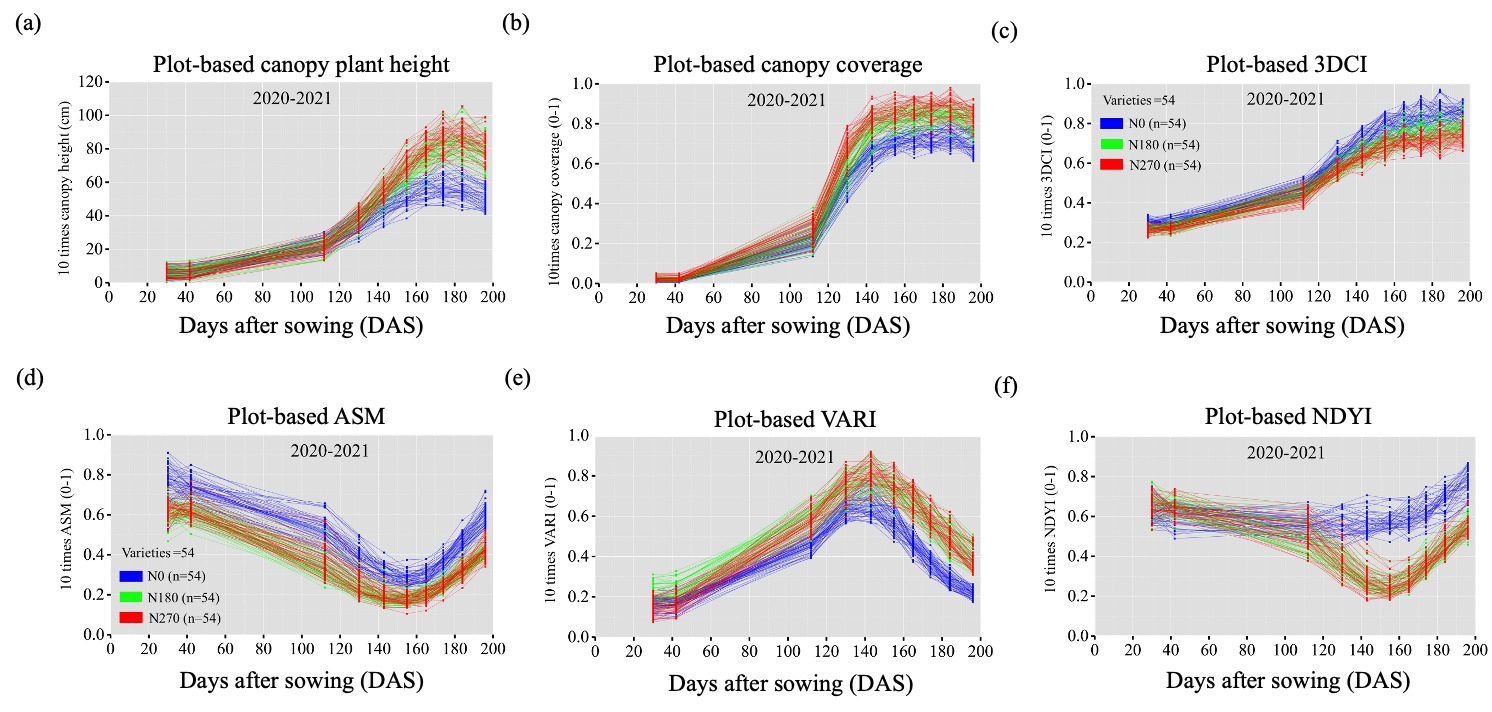


(a-f) The rough changing profiles of plot-based canopy plant height, canopy coverage, 3DCI, ASM, VARI and NDYI based on 10 times phenotypic analysis results using the AirMeasurer platform.

**Figure S4.** The complete CCR matrix created to represent 5-day phenotypic changes of six selected traits (n = 972 plots, two seasons) after the jointing N fertilization, which was used to dissect N-response phenotypic changes. Pseudo-color applied to highlight changes according to a scale bar (<-12.5% – >12.5%).


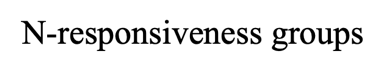

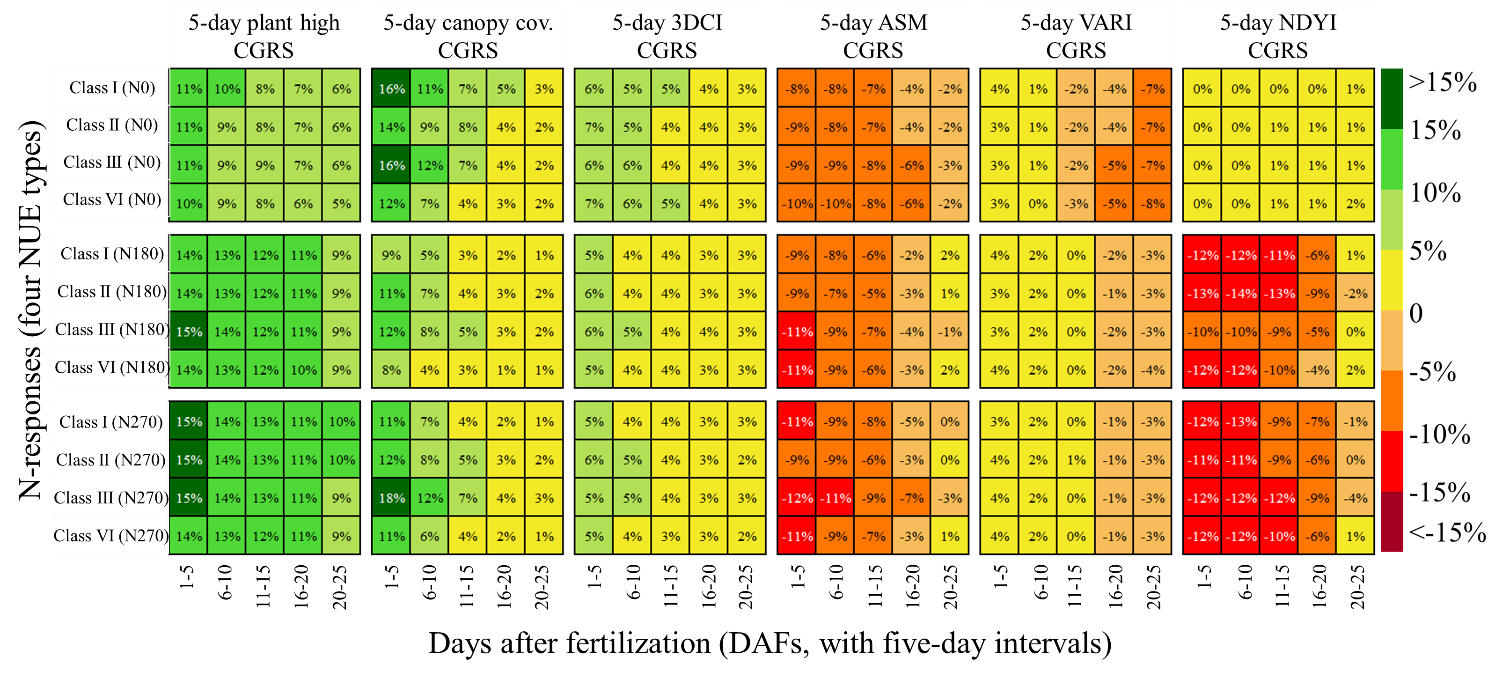


**Figure S5**. The SNP density map, population structure, and GWAS analysis results using static and dynamic phenotypes of N-response related traits, which were estimated by the AirMeasurer platform and field specialists.


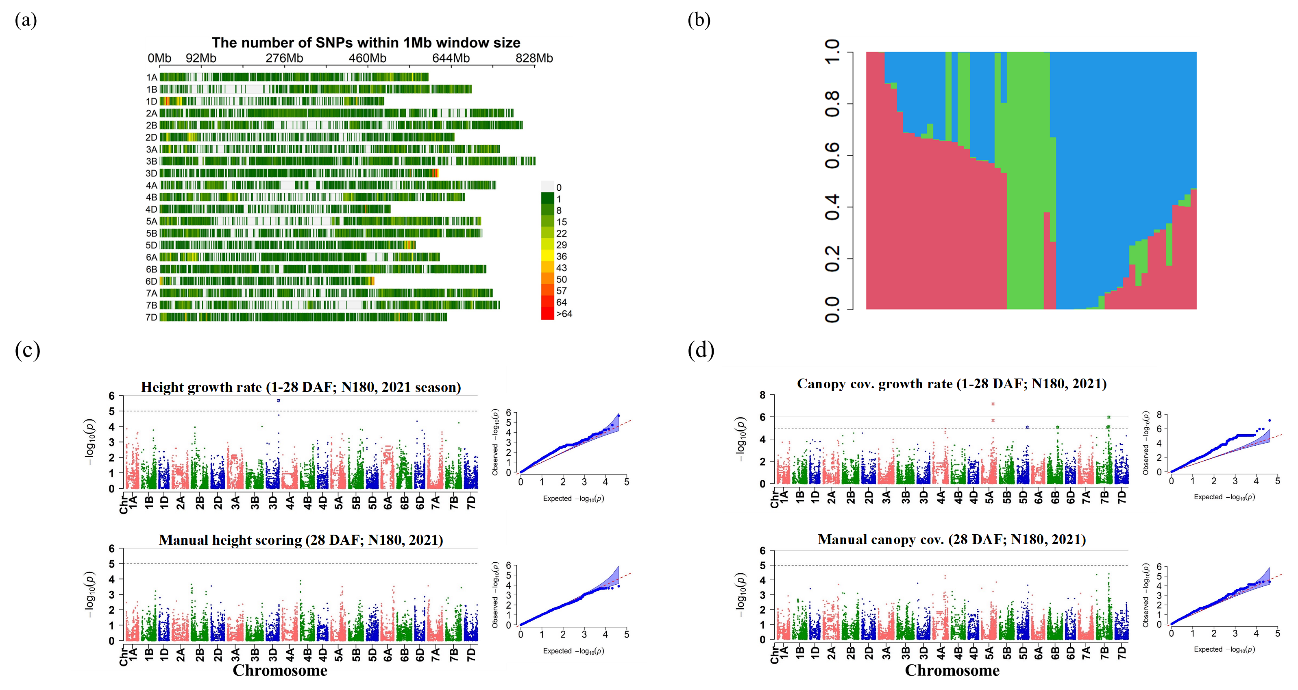


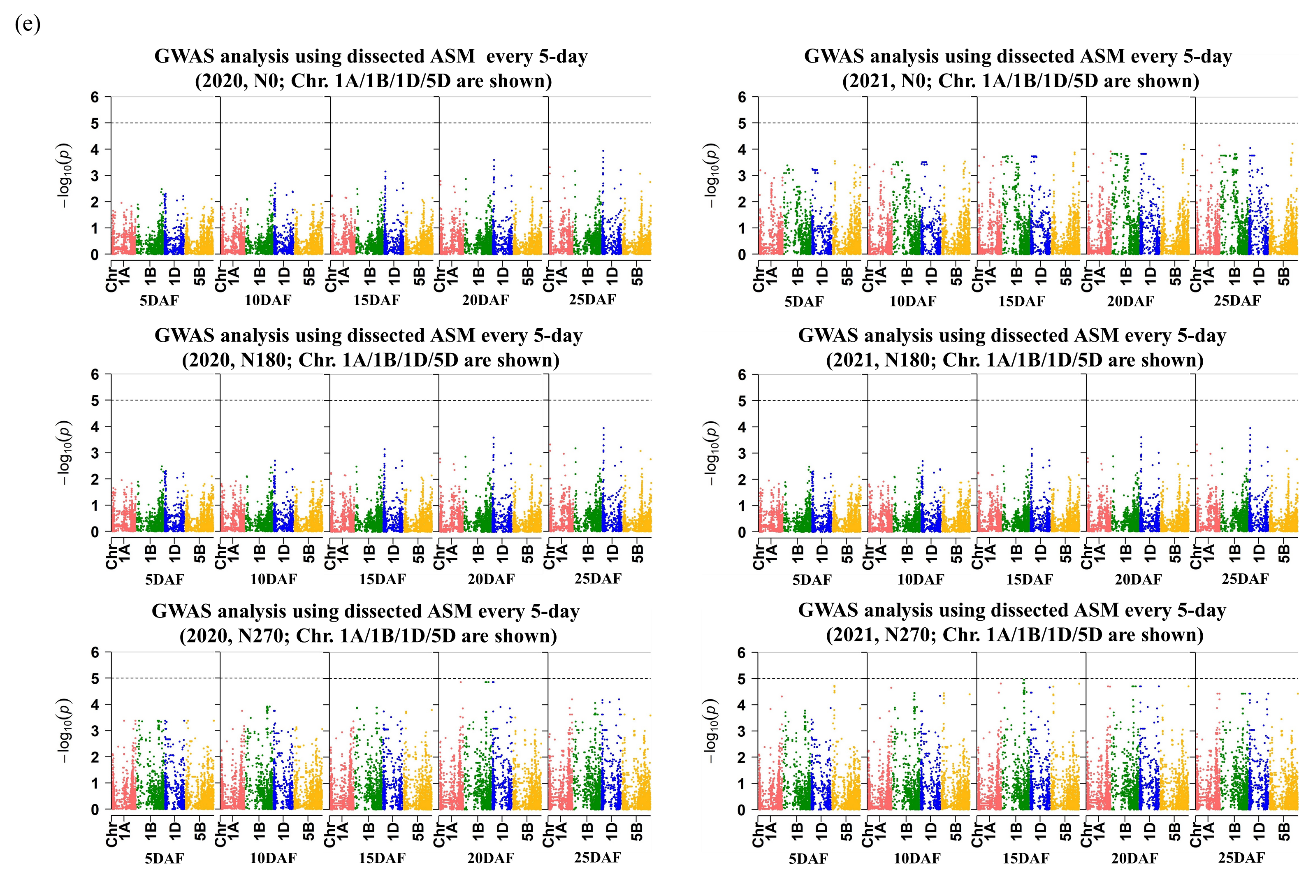


(a) Chromosomal distribution of SNP markers mapping with the 54 varieties. (b) Analysis of the population structure. (c) GWAS results using the change rate of plant height manually measured and computationally estimated. (d) GWAS using canopy coverage manually measured and computationally estimated. (e) GWAS results using ASM with five-day intervals.

**Figure S6.** Two-season profile curves of six N-response related traits using morphological, textural, spectral signals collected by drone phenotyping across 2019-2021 seasons.


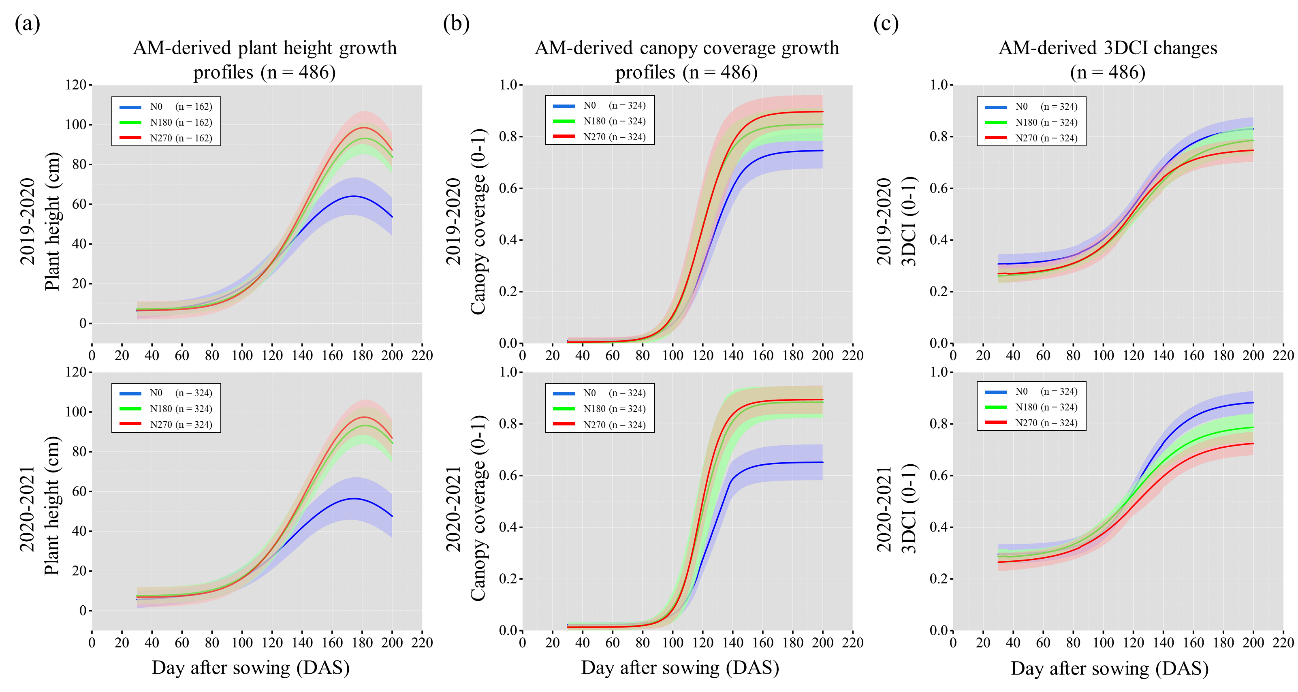


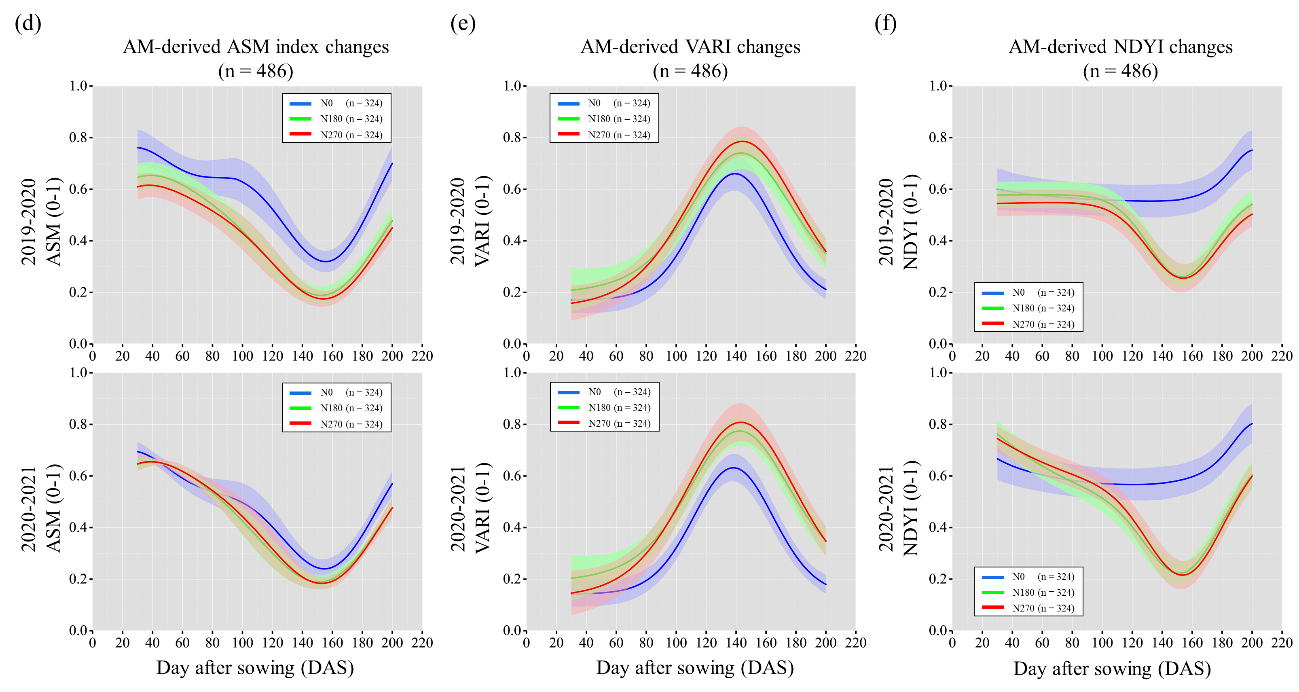


(a) The two-season plant height profile curves produced using 486 plots under three N treatments. (b-f) The two-season profile curves for canopy coverage, 3DCI, ASM, VARI and NDYI.

**Note S1** Principal component analysis (PCA) used for classifying N-responsiveness groups

The comprehensive evaluation scores of different varieties were calculated based on the variance contribution rate, feature weight vector, and principal component values. The calculation formula is listed as follows:

$$\begin{aligned} {PC}_{ji}=X_{i}\Phi_{j}\#\left( 1 \right) \end{aligned}$$

Where *PC*_𝑗𝑖_ is the principal component (PC) value of the *j^th^* component of the *i^th^* variety, 𝑋_𝑖_ is the index value of the *i^th^* sample after standardization, and *Φ_j_* is the feature weight vector of the trait of the *j^th^* component.

Under three levels of N treatments, the top four PCs with cumulative variance contribution rate greater than 85% was computed using the following equations:

${Score}_{N0}=33.6\%*{PC}_{1}+23.7\%*{PC}_{2}+18.0\%*{PC}_{3}+ 12.4\%*{PC}_{4}$ (2)

${Score}_{N180}=35.2\%*{PC}_{1}+23.6\%*{PC}_{2}+16.5\%*{PC}_{3}+ 11.9\%*{PC}_{4}$ (3)

${Score}_{N270}=40.6\%*{PC}_{1}+20.8\%*{PC}_{2}+18.6\%*{PC}_{3}+ 10.3\%*{PC}_{4}$ (4)

Where ${PC}_{1-4}$ are scores of every PC, with a combined 80% cumulative contribution rate; coefficient of every PC is the variance contribution rate.

**Note S2** Weight calculation of the N-response traits

The weights $W_{p,t}$ were calculated for the six N-response traits based on the results of PCA for every five-day phase during 1-25 day after fertilization (DAF), under three N treatments. The weights $W_{p}$ were calculated using the equation:

$W_{p}=\sum_{t=1}^{6} W_{p,t}$ (5)

According to the six N-response traits’ phenotypic changes, the PCA was performed during1-25DAF, and the weights $TW_{p,t}$ of the six traits in each 5-day phase were calculated, and then the combined weight $W_{t}$ of each trait in the five stages was calculated using the equation:

$W_{t}={TW}_{p,t}\bullet W_{p}$ (6)

Where $p$ is 1-5, denoting five phases (e.g. 1-5 DAF, 6-10 DAF, 11-15 DAF, 16-20 DAF and 21-25 DAF), respectively; $t$ is 1-6, denoting the six target traits such as height, coverage, 3DCI, ASM, VARI and NDYI, respectively.

**Note S3** A Random Forest based model to classify N responsiveness groups in wheat

The Random Forest (RF) model randomly generates a number of independent decision trees using the bagging sampling method, each of which outputs the category of N responsiveness (i.e. N-dependent yield increase). The final result of the model is determined by the Majority Vote Taken (MVT) algorithm. In our case, the most important step in the generation of the optimized RF-NRES model was how to select the optimal division parameters of the branch nodes. Since phenotypic changes of N-response related traits has different weights in connection with yield performance and N utilization, the parameters were calculated using principal component analysis (PCA) derived weights and then employed as hyperparameters of the RF-NRES model. When calculating the information gain of a decision node and selecting the optimal parameter, we multiply the information gain value of each parameter by the corresponding PCA-derived weight. The equation used is listed as follows:

$$\begin{aligned} Ent\left( D \right)=-\sum_{k}^{5} p_{k}{log}_{2}p_{k}\#\left( 7 \right) \end{aligned}$$

The information entropy *Ent(D)* in *D* denotes the data set of the current decision node, and $p_{k}$ denotes the proportion of the *k* class of samples in the set.

$$\begin{aligned} IG\left( D,t \right)=Ent\left( D \right)-\sum_{v=1}^{V} \frac{{|D}^{v}|}{\left| D \right|}Ent\left( D^{v} \right)\#\left( 8 \right) \end{aligned}$$

The information gain function, $IG\left( D,t \right)$, is listed above, where *t* denotes a given N-response trait and *V* denotes the number of branch nodes. Using *t* trait to divide the sample *D* generates *V* branches, where the set of samples of the *v* branch is $D^{v}$.
